# Validation of a German version of the Cerebellar Cognitive Affective/ Schmahmann Syndrome Scale: preliminary version and study protocol

**DOI:** 10.1186/s42466-020-00071-3

**Published:** 2020-09-29

**Authors:** Andreas Thieme, Sandra Roeske, Jennifer Faber, Patricia Sulzer, Martina Minnerop, Saskia Elben, Heike Jacobi, Kathrin Reetz, Imis Dogan, Miriam Barkhoff, Juergen Konczak, Elke Wondzinski, Mario Siebler, Oliver Mueller, Ulrich Sure, Jeremy D. Schmahmann, Thomas Klockgether, Matthis Synofzik, Dagmar Timmann

**Affiliations:** 1 Department of Neurology, Essen University Hospital, University of Duisburg-Essen, Hufelandstr. 55, 45147 Essen, Germany; 2 German Center for Neurodegenerative Diseases (DZNE) Bonn, Helmholtz Association, Venusberg-Campus 1, 53127 Bonn, Germany; 3 Department of Neurology, Bonn University Hospital, Rheinische Friedrich-Wilhelms University Bonn, Venusberg-Campus 1, 53127 Bonn, Germany; 4 Department of Neurodegenerative Diseases, Hertie-Institute for Clinical Brain Research and Center of Neurology, Eberhard Karls University Tuebingen, Hoppe-Seyler-Str. 3, 72076 Tuebingen, Germany; 5 German Center for Neurodegenerative Diseases (DZNE) Tuebingen, Helmholtz Association, Otfried-Mueller-Straße 23, 72076 Tuebingen, Germany; 6 Institute of Neuroscience and Medicine (INM-1), Research Centre Juelich, Wilhelm-Johnen-Str., 52425 Juelich, Germany; 7 Department of Neurology, Center for Movement Disorders and Neuromodulation, Medical Faculty, Heinrich-Heine University Duesseldorf, Moorenstr. 5, 40225 Duesseldorf, Germany; 8 Institute of Clinical Neuroscience and Medical Psychology, Medical Faculty, Heinrich-Heine University Duesseldorf, Moorenstr. 5, 40225 Duesseldorf, Germany; 9 Department of Neurology, Heidelberg University Hospital, Ruprecht-Karls University Heidelberg, Im Neuenheimer Feld 400, 69120 Heidelberg, Germany; 10 Institute of Neuroscience and Medicine (INM-11), Research Centre Juelich, Wilhelm-Johnen-Str., 52425 Juelich, Germany; 11 Department of Neurology, Aachen University Hospital, Rheinisch-Westfaelische Technische Hochschule Aachen (RWTH), Pauwelstr. 30, 52074 Aachen, Germany; 12 JARA-BRAIN Institute, Molecular Neuroscience and Neuroimaging, Research Centre Juelich, Wilhelm-Johnen-Str., 52425 Juelich, Germany; 13 School of Kinesiology, University of Minnesota, 400 Cooke Hall 1900 University Ave S E, Minneapolis, MN 55455 USA; 14 Department of Neurology and Neurorehabilitation, MediClin Fachklinik Rhein/ Ruhr, Auf der Roetsch 2, 45219 Essen, Germany; 15 Present Address: Department of Neurosurgery, Klinikum Dortmund, Muensterstr. 240, 44145 Dortmund, Germany; 16 Department of Neurosurgery, Essen University Hospital, University of Duisburg-Essen, Hufelandstr. 55, 45147 Essen, Germany; 17 Department of Neurology, Ataxia Center, Cognitive Behavioral Neurology Unit, Laboratory for Neuroanatomy and Cerebellar Neurobiology, Massachusetts General Hospital, Harvard Medical School, 55 Fruit Street, Boston, MA 02114 USA

**Keywords:** Affect, Cerebellum, Bedside test, Cognition, Human

## Abstract

**Background:**

Traditionally, cerebellar disorders including ataxias have been associated with deficits in motor control and motor learning. Since the 1980’s growing evidence has emerged that cerebellar diseases also impede cognitive and affective processes such as executive and linguistic functions, visuospatial abilities and regulation of emotion and affect. This combination of non-motor symptoms has been named *Cerebellar Cognitive Affective/ Schmahmann Syndrome (CCAS)*. To date, diagnosis relies on non-standardized bedside cognitive examination and, if available, detailed neuropsychological test batteries. Recently, a short and easy applicable bedside test (CCAS Scale) has been developed to screen for CCAS. It has been validated in an US-American cohort of adults with cerebellar disorders and healthy controls. As yet, the CCAS Scale has only been available in American English. We present a German version of the scale and the study protocol of its ongoing validation in a German-speaking patient cohort.

**Methods:**

A preliminary German version has been created from the original CCAS Scale using a standardized translation procedure. This version has been pre-tested in cerebellar patients and healthy controls including medical experts and laypersons to ensure that instructions are well understandable, and that no information has been lost or added during translation. This preliminary German version will be validated in a minimum of 65 patients with cerebellar disease and 65 matched healthy controls. We test whether selectivity and sensitivity of the German CCAS Scale is comparable to the original CCAS Scale using the same cut-off values for each of the test items, and the same pass/ fail criteria to determine the presence of CCAS. Furthermore, internal consistency, test-retest and interrater reliability will be evaluated. In addition, construct validity will be tested in a subset of patients and controls in whom detailed neuropsychological testing will be available. Secondary aims will be examination of possible correlations between clinical features (e.g. disease duration, clinical ataxia scores) and CCAS scores.

**Perspective:**

The overall aim is to deliver a validated bedside test to screen for CCAS in German-speaking patients which can also be used in future natural history and therapeutic trials.

**Study registration:**

The study is registered at the German Clinical Study Register (DRKS-ID: DRKS00016854).

## Background

Cerebellar disease results in well-known motor performance deficits, including ataxia of stance and gait, limb incoordination, dysarthria, and oculomotor abnormalities. During the last decades, there has been growing evidence that cerebellar disease is not only accompanied by motor disturbances but also by cognitive and affective symptoms (see [1–3] for reviews). As early as 1998, Schmahmann and Sherman introduced the *Cerebellar Cognitive Affective/ Schmahmann Syndrome (CCAS)* [4]*.* The core symptoms of CCAS are difficulties with executive, linguistic and visuospatial functions as well as problems with the regulation of emotion and affect. Since its original description, evidence for the presence of CCAS has been accumulating in pediatric and adult patients suffering from different cerebellar diseases including various hereditary ataxias, cerebellar tumors, and cerebellar stroke [1–3]. In recent years, advances in structural and functional brain imaging allowed for detailed mapping of cognitive functions in the posterolateral cerebellar hemisphere [5–9]. As yet, diagnosis of CCAS relies on non-standardized bedside cognitive examination and, if available, detailed neuropsychological test batteries. Until recently, there has not been a validated bedside test that was able to reliably screen for CCAS in cerebellar patients – unlike well-established bedside tests for dementias or mild cognitive impairment (MCI), i.e. Mini Mental State Examination (MMSE) or Montreal Cognitive Assessment (MoCA). However, MMSE and MoCA are of limited use to screen for CCAS because cerebellar patients frequently perform within the normal range [10]. Recently, Schmahmann and colleagues [10] have developed a bedside test designed to screen for CCAS in adults. In order to develop this CCAS Scale, they first applied a broad battery of 36 well-established neuropsychological tests in a large group of cerebellar patients primarily suffering from cerebellar degeneration. In the novel CCAS Scale, tests were implemented which captured the core cognitive domains of CCAS, distinguished best between cerebellar patients and controls, and at the same time were short and easy enough to be applied in a bedside setting. These include test items for semantic and phonemic fluency, category switching, verbal registration and delayed verbal recall, digit span forward and backward, cube draw and copy, similarities, go/ no-go, and affect. Single tests can either be passed reaching a specific cut-off score or failed. CCAS is considered possible if one test is failed, probable if two tests are failed, and definite if three or more tests are failed. Version A of the CCAS Scale has then been validated in another US-American cohort of 39 adult cerebellar patients, including patients with cerebellar degeneration and focal cerebellar lesions and 55 matched healthy controls. It exhibited high values for selectivity [that is the ability to distinguish between patients and controls, or in other words preventing controls from being diagnosed as patients; possible/ probable/ definite CCAS: 78/ 93/ 100%] and reasonable sensitivity [that is the probability that a patient is identified as a patient; possible/ probable/ definite CCAS: 95/ 82/ 46%]. Furthermore, it showed modest internal consistency using Cronbach’s alpha value (= 0.59) indicating that no test item within the scale measures the exact same domain(s) as another item. Thus, no test item is redundant. Comparing patients with pure cerebellar lesions and patients with additional extracerebellar involvement the authors found that difficulties in verbal registration and delayed verbal recall were more prominent in the latter. Therefore, poor performance in these two test items is indicative of extracerebellar involvement (“red flag”). In addition to the pass/ fail criteria which are used to screen for CCAS, a total sum score is calculated which allows for follow-up examinations in individual patients [10]. Three parallel versions B-D were developed to enable repeated assessments. In the present study preliminary German versions of the CCAS Scale are introduced, and the study protocol for their validation is presented.

## Methods

### Study aims

The first primary aim of this study was to create preliminary German versions of the CCAS Scale. The second primary aim will be their validation in a large cohort of patients with various cerebellar disorders and healthy age-, sex-, and education-matched controls. Secondary aims will be to examine possible relationships between clinical features such as disease duration or severity of cerebellar motor symptoms, and the CCAS score.

### Study description and study design

#### Translation process of the original CCAS Scale into German language

A group of medical experts translated the original American English versions A-D of the CCAS Scale into German accounting for language- (and cultural-) dependent differences while staying as close as possible to the original CCAS Scale. A standardized, six step procedure was used following guidelines for cross-cultural translation, adaptation, and validation of self-report measures, instruments, or scales for use in healthcare research [11, 12]. The expert group comprised three independent teams each consisting of two individuals (team 1: University Hospital and German Center for Neurodegenerative Diseases Bonn: S. Roeske = neuropsychologist, J. Faber = neurologist; team 2: University Hospital Essen: D. Timmann, A. Thieme = neurologists; team 3: University Hospital Tuebingen: P. Sulzer = neuropsychologist, M. Synofzik = neurologist).

**Fig. 1:**
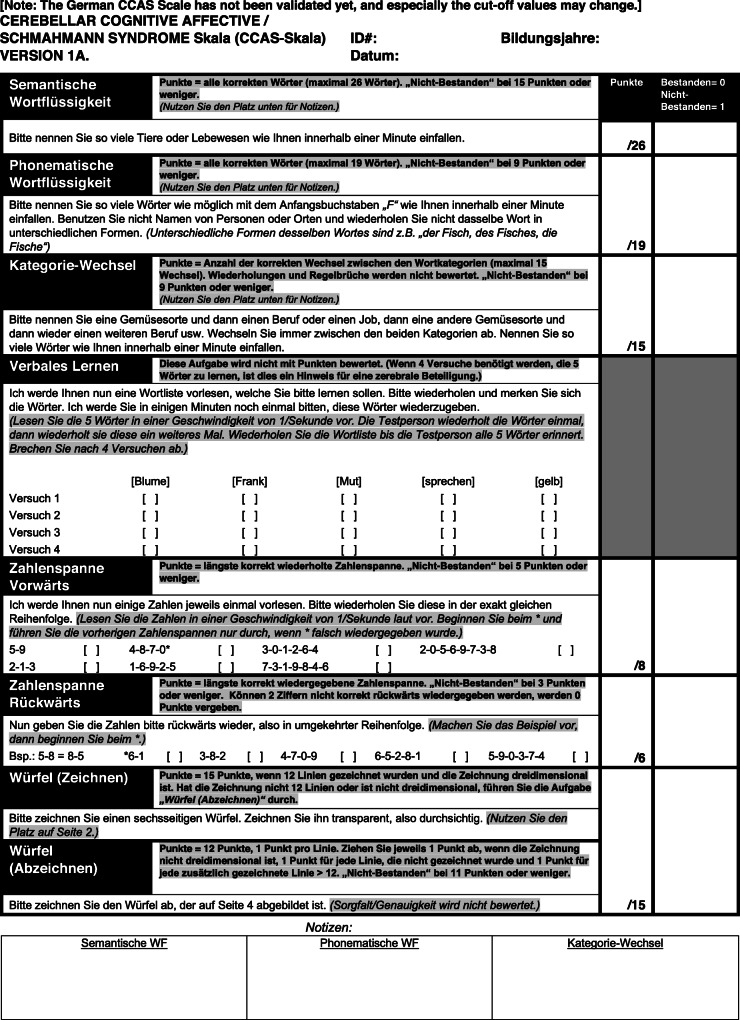

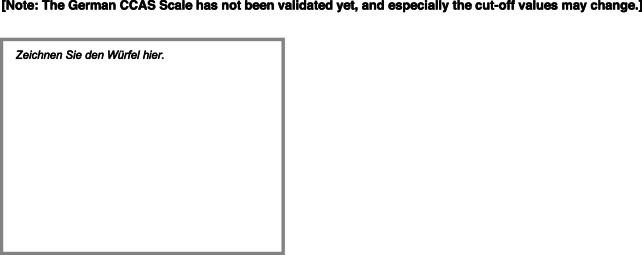

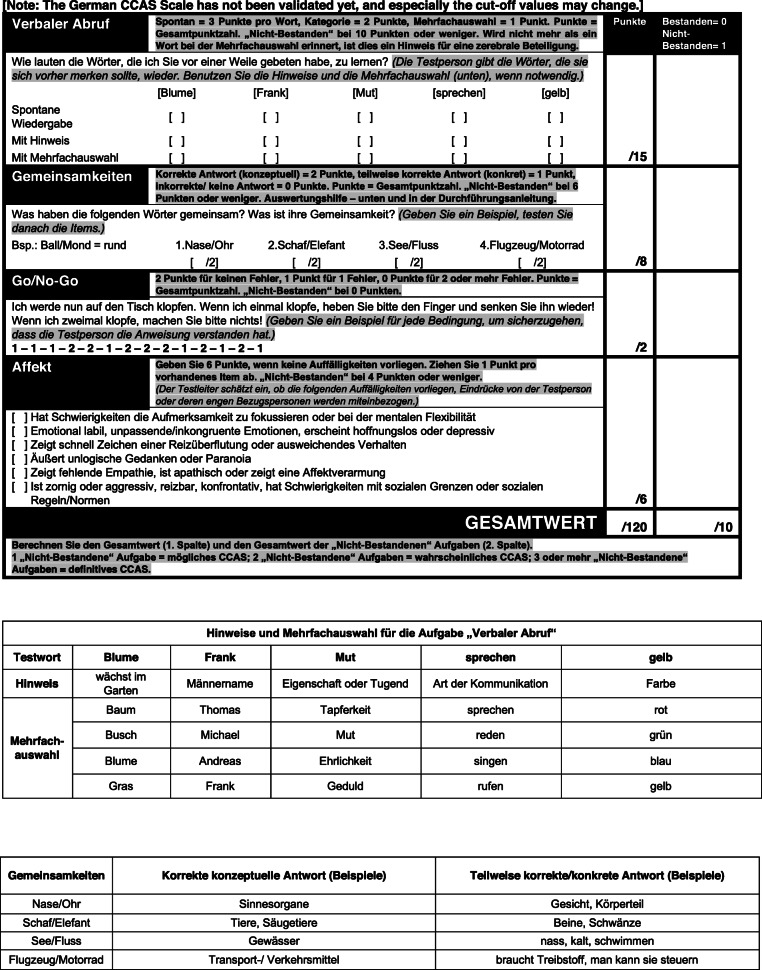

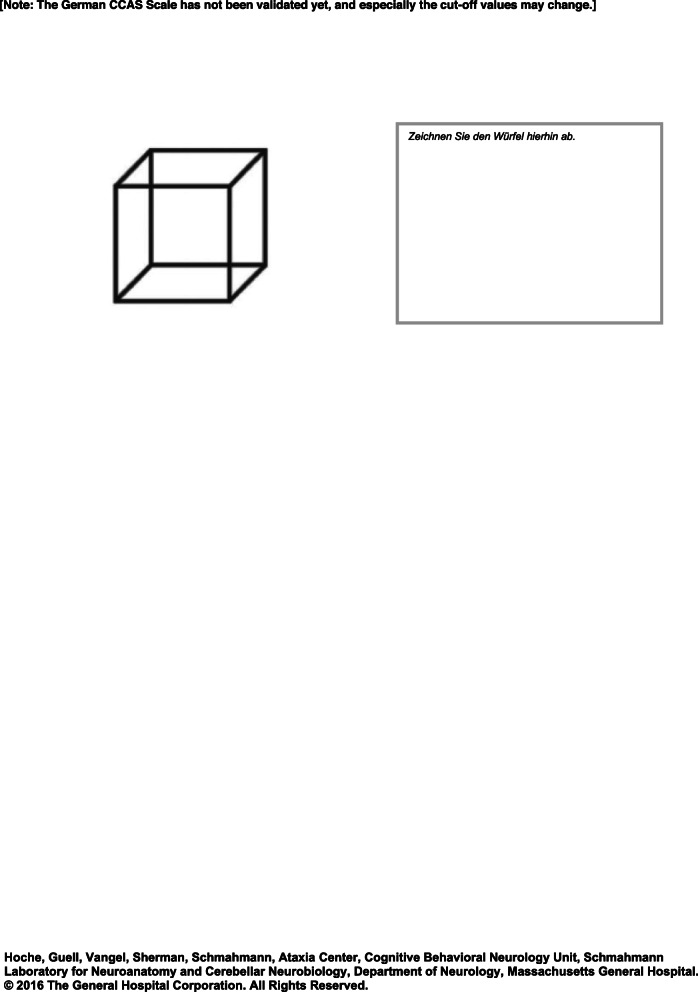
Preliminary German CCAS Scale, Version A

*Step 1:* Each team translated the original CCAS Scales (A-D) independently to German. *Step 2:* For each version (A-D) a consensus version was derived. *Step 3:* Consensus version A was then translated back to American English by a bilingual expert (J. Konczak = neuroscientist). The parallel versions B-D were not translated back because instructions on the test form were similar in all versions. *Step 4:* Discrepancies were resolved in a joined discussion and a German prototype version A was formed. The senior author (J. D. Schmahmann) of the original CCAS Scale was involved in this step (and step 6, see below) to ensure that no information has been lost or added during the translation process. *Step 5:* The prototype version A was pretested in a small cohort of medical experts and laypersons. The medical expert group consisted of eleven neurologists, three neuropsychologists and one medical student (mean age: 33.8 ± 6.4 yrs.; age range: 23.8–49.0 yrs.; 6 males, 9 females; mean education: 19.6 ± 1.2 yrs.). None of them was involved in steps 1–4. The lay group consisted of 12 cerebellar patients, one healthy subject and three healthy first-degree relatives of patients with hereditary ataxias, i.e. persons at risk (mean age: 59.5 ± 14.9; age range: 23.4–84.0 yrs.; 8 males, 7 females; mean education: 15.2 ± 4.2 yrs.). All participants were asked to rate each item of the German prototype version A as *“easy to understand”*, *“comprehensible”*, *“difficult to understand”*, *or “incomprehensible”*. *Step 6:* Imprecise and misleading items were revised (for details see Supplementary Materials, Part 2). The resulting preliminary version of the CCAS Scale, Version A, is shown in Fig. 1. The parallel versions were revised accordingly and are shown in Supplementary Materials, Part 3.

There are also detailed test instructions for the examiner. Firstly, these instructions have been translated independently by each team, and next a consensus was derived (see Supplementary Materials, Part 4).

#### Validation process

##### Inclusion and exclusion criteria

To be eligible for the study, subjects must be 18 years or older, German-speaking (primary language) and they must be able to understand and follow instructions and to give informed consent. Exclusion criteria comprise neurological or psychiatric disorders in control participants, and neurological disease other than cerebellar disease and primary psychiatric disorders in patients. Patients will be included who suffer from degenerative cerebellar disorders or focal cerebellar lesions (e.g. cerebellar stroke, tumor, or cerebellar surgical lesions). Alcohol or drug abuse, intake of centrally acting drugs (other than low dose antidepressants) and consuming diseases or general poor health are further exclusion criteria for all participants. Furthermore, participants under legal supervision will not be recruited. For detailed in- and exclusion criteria see Table 1.

**Table 1:** Inclusion and exclusion criteria for patient and control selection

Group	Inclusion criteria	Exclusion criteria
**General**	- Age ≥ 18 years - German-speaking (primary language) - Informed consent	- Alcohol or drug abuse - Intake of centrally acting drugs (other than low-dose antidepressants) - Consuming diseases - Poor health condition - Persons under legal supervision
**Patients**	- General inclusion criteria	- General exclusion criteria - Severe primary psychiatric disorders
**Degenerative Cerebellar Disorders**		
**Cer-pure** Disorders primarily affecting the cerebellum	- SAOA - EA1 and EA2 - SCA6 - SCA8 - SCA14 - ANO10 - Post-inflammatory cerebellar degeneration	
**Cer-plus** Disorders with relevant extracerebellar involvement	- MSA-C - all other hereditary ataxias including: SCA1, 2, 3, Friedreich’s ataxia, early onset cerebellar ataxias	
**Focal Cerebellar Lesions**		
**Cer-pure/ cer-plus**	- Cerebellar stroke - Cerebellar hemorrhage - Cerebellar tumor - Cerebellar surgical lesion	
**Controls**	- General inclusion criteria	- General exclusion criteria - Neurological and psychiatric disorders
**Pretesting Subjects**	- General inclusion criteria	- General exclusion criteria - Neurologists and psychologists that were involved in the development of the German CCAS Scales

*Abbreviations*: *Cer-pure* Isolated Cerebellar Disease/ Lesion, *Cer-plus* Cerebellar Plus Disease/ Lesion, *SAOA* Sporadic Adult Onset Ataxia, *EA1* Episodic Ataxia Type 1, *EA2* Episodic Ataxia Type 2, *SCA1, 2, 3, 6, 8, 14* Spinocerebellar Ataxia Type 1, 2, 3, 6, 8, 14, *ANO10* Spinocerebellar Ataxia, Autosomal-Recessive Type 10, *MSA-C* Multisystem Atrophy Cerebellar Type, *CCAS* Cerebellar Cognitive Affective/ Schmahmann Syndrome

##### Study sample

For validation of version A at least 65 patients and an equal number of matched healthy controls will be recruited. About one third (≥ 25) of the patients will suffer from disorders that primarily affect the cerebellum (isolated cerebellar disease = cer-pure: 1. degenerative cerebellar disorders, e.g. spinocerebellar ataxia type 6 (SCA6); 2. focal cerebellar lesions, e.g. cerebellar stroke). The other two thirds (≥ 40 patients) will suffer from disorders with additional extracerebellar involvement (cerebellar plus disease = cer-plus: 1. degenerative cerebellar disorders, e.g. SCA1, 2 or 3; 2. focal cerebellar lesions, e.g. cerebellar stroke with additional involvement of the brain stem). Group assignment in patients with cerebellar degeneration goes by diagnosis: A genetic disease, which is known to involve extracerebellar regions, is considered a “cer-plus form”, although the clinical phenotype at the time of testing may be pure cerebellar.

An equivalent number of healthy matched participants will serve as controls. Each patient will be matched with a control participant of same sex, similar age (interval: +/− 5 years) and similar years of education (intervals: < 9 yrs., 9–10 yrs., 11–13 yrs., 14–16 yrs., > 16 yrs.). We will use the same matching criteria as in [10] except for education matching because of the different educational systems in Germany and the United States.

Parallel versions B-D will be validated in groups of 25 patients (cer-pure and cer-plus, respectively) and 25 matched healthy controls each.

Patients in this investigator-initiated, multicenter study will be recruited from the ataxia clinics at the Departments of Neurology of the University Hospitals in Aachen, Bonn, Duesseldorf, Essen, Heidelberg and Tuebingen, as well as from the MediClin Fachklinik Rhein-Ruhr in Essen, and the Departments of Neurosurgery of the University Hospital in Essen and at the Klinikum Dortmund. Essen is the coordinating site. Furthermore, we will collaborate with the “Deutsche Heredo-Ataxie Gesellschaft e.V. (DHAG)”, a patient support group, and patients will be recruited via their newsletter and webpage. Healthy controls will be recruited from patients’ families and by public bulletins.

##### Demographics and clinical assessment of cerebellar motor syndrome

Demographics including years of education and employment, educational achievements as well as occupational and marital status will be recorded. For patients, medical records and available brain scans will be evaluated. Age of onset, disease duration and in case of genetically proven nucleotide repeat diseases the repeat length will be documented. Furthermore, a detailed medical history will be taken, and a neurological examination will be performed in every participant. Severity of cerebellar ataxia in patients will be evaluated using different clinical ataxia scales: The Scale for the Assessment and Rating of Ataxia (SARA) [13] will be used because of its widespread use. In addition, the International Cooperative Ataxia Rating Scale (ICARS) [14] will be used because it includes rating of cerebellar oculomotor deficits. Its short form – BARS – [15] will be used to enable direct comparison with the original US-American validation study. The SpinoCerebellar Ataxia Functional Index (SCAFI) [16] will be assessed because it allows for a more objective quantification of motor deficits. Finally, non-ataxia signs will be assessed semi-quantitatively using the Inventory of Non-Ataxia Signs (INAS) [17].

##### Assessment of the CCAS Scale

The CCAS Scale will be administered in each participant at least once. In the validation process of version A, at least 40 patients and 40 controls will receive a follow-up examination with the same version of the scale to determine test-retest and interrater reliability (≥ 20 patients and ≥ 20 controls, respectively). Retesting will be done with the same version because equivalence of versions A-D has not yet been shown (see [10] and Supplements). Follow-up will take place within an interval of 14 to 56 days. A time interval is favored instead of a fixed time span between test and retest (e.g. exactly 14 days) to control for learning effects by correlating retest results with different retest time intervals.

To assess construct validity of the German version of the CCAS Scale a detailed neuropsychological testing will be done in a subset of patients (*n* ≥ 20) and controls (*n* ≥ 20) using well-established neuropsychological test batteries available in German. These will comprise the logical memory test (part I and II) of the Wechsler Memory Scale – 4th edition (WMS-IV) [18], the copy immediate and delayed recall of the Rey–Osterrieth Complex Figure Test (ROCFT) [19], and the letter-number sequencing task of the WAIS-IV [20]. These tests measure the same cognitive domains as corresponding items of the CCAS Scale. Furthermore, the German version 3 of the MoCA [21] will be assessed for direct comparison with the CCAS Scale. Finally, the Patient Health Questionnaire (PHQ-9) [22] and the German version of the EuroQuol – 5 dimension – 3 level (EQ-5D-3L) questionnaire [23] will be administered.

Validation of parallel versions of the CCAS Scale will be done the same way as for validation of parallel German versions of the MoCA [21]. Each parallel version (B/C/D) of the CCAS Scale will be tested against version A. Testing of version B/C/D and version A will be done on the same day, with the order being randomized between participants.

##### Data analysis

Selectivity and sensitivity will be assessed using the same cut-off values for individual test items, and the same three pass/ fail criteria determined in the original study by Hoche et al. [10] (that is: possible CCAS = one test failed; probable CCAS = two tests failed; definite CCAS = three or more tests failed). To assess selectivity the percentage of true negatives will be calculated, that is the percentage of controls which have been correctly identified as controls [number of controls identified as controls/ true number of controls in the sample * 100]. To assess sensitivity the percentage of true positives will be calculated, that is the percentage of patients which have been correctly identified as patients [number of patients identified as patients/ true number of patients in the sample * 100]. In case selectivity and sensitivity falls below the values of the original CCAS Scale, selectivity and sensitivity of individual tests will be assessed. Cut-off values of single test items and/ or cut-offs defining (possible/ probable/ definite) CCAS will be adjusted to achieve high selectivity and reasonable sensitivity – comparable to the values of the original CCAS Scale.

Differences between patient groups (pure cerebellar disease vs. patients with additional extracerebellar involvement) will also be analyzed. We want to verify that difficulties in verbal registration and delayed verbal recall are indicative of extracerebellar involvement (“red flags”). In further accordance with Hoche et al. [10], Cronbach’s alpha will be used to assess the inter-relatedness of the individual test items, i.e. internal consistency.

To study construct validity of the CCAS Scale subtests of validated German versions of neuropsychological test batteries will be used as the external criterion. We will compare the percentage of patients diagnosed with CCAS based on a detailed neuropsychological test battery with the percentage of patients identified by the CCAS Scale.

Finally, correlations between age, disease duration, severity of cerebellar motor symptoms (measured by clinical ataxia scores), and total CCAS sum score will be calculated. All raw data (thus total number of failed tests respective total sum score and sub scores on single test items) will be tested for normal distribution using Kolmogorov-Smirnov tests. Parametric or non-parametric tests will be applied depending on distribution and final sample size.

## Perspective

Clinical ataxia scales have been validated to rate the severity of motor symptoms in cerebellar disease [13–17], but so far there has not been a validated clinical scale to screen for the presence of the Cerebellar Cognitive Affective/ Schmahmann Syndrome (CCAS), and quantify CCAS severity. Only recently, Schmahmann and collaborators have developed and validated a promising screening tool for the CCAS – the CCAS Scale – for an American English-speaking population [10]. Till now, no validated German versions of the scale exist. German versions of the CCAS Scale are highly desirable to screen for the presence of CCAS in a clinical setting, but also for patient characterization and stratification in future therapeutical trials. The CCAS sum score may also serve as a therapeutic marker, but this would need future studies to show its sensitivity to change, and if this would be the case, its treatment responsiveness. In this multicenter study, four parallel versions (A-D) of the German CCAS Scale will be validated in a large cohort of German-speaking patients with cerebellar disorders. The parallel versions will allow multiple testing without practice effects.

The primary aim of our validation study is to show high selectivity, that is to show that the German versions of the scale are able to differentiate between patients and controls. We will test whether the pass/ fail criteria used to determine the presence of CCAS in the original scale also apply for the German scale. Cut-off values of individual test items and the pass/ fail criteria shown in Fig. 1 are taken from the US-American original and may change depending on findings in the German validation cohort. Furthermore, future studies are needed to test for possible age effects, and the need for age-dependent cut-off values and pass/ fail criteria. After full validation of the scale the implementation of a web-based training tool is planned.

We are interested whether the scale is able to screen for CCAS in patients with degenerative diseases, but also suffering from cerebellar stroke or surgical lesions due to cerebellar tumors. In the validation study of the original American English CCAS Scale only few patients with focal cerebellar lesions were tested [10]. In part of the patients with focal lesions brain MRI scans will be available. This will allow to map dysfunction in the different cognitive domains to lesions in specific cerebellar regions. For example, we expect that language dysfunction is associated with lesions of the right posterolateral cerebellar hemisphere, and visuospatial disabilities with lesions of the left posterolateral cerebellar hemisphere [6].

A limitation of the CCAS Scale is that detection of the neuropsychiatric abnormalities highly depends on the examiner’s expertise. The authors of the US-American original were aware of this weakness and gave this item (“Affect”) a weak denominator for the total sum score [10]. Additionally, administration of more detailed scales of neuropsychiatric dysfunction are recommended [24]. In future studies, expert neuropsychiatric assessments would be of interest as a further external criterion. Furthermore, testing of construct validity has some limitations. We will perform detailed neuropsychological testing only in a subset of patients. More importantly, although the core symptoms of CCAS are well described, as yet there are no standard criteria to diagnose CCAS based on detailed neuropsychological testing. Another approach to test construct validity would be to compare CCAS scores in patients with cerebellar diseases and patients with non-cerebellar neurological diseases in the future. Despite these limitations the CCAS Scale has been shown to be more sensitive to detect cognitive and affective changes in cerebellar disease than the Mini-Mental State Examination and the Montreal Cognitive Assessment [10]. The validated German CCAS Scale will allow trained healthcare personnel to screen for cognitive and affective symptoms in patients with cerebellar diseases in German-speaking countries.

## Supplementary information

**Additional file 1.**

## Data Availability

The datasets used to analyse the pretesting experiment are available from the corresponding author on reasonable request.

## Abbreviations

ADHD: Attention Deficit Hyperactivity Disorder
ANO10: Spinocerebellar Ataxia, Autosomal-Recessive Type 10
BARS: Brief Ataxia Rating Scale
CCAS: Cerebellar Cognitive Affective/ Schmahmann Syndrome
CNRS: Cerebellar Neuropsychiatric Rating Scale
DFG: Deutsche Forschungsgemeinschaft
D-KEFS: Delis-Kaplan Executive Function System
DRKS: Deutsches Register Klinischer Studien
EA1/2: Episodic Ataxia Type 1/2
Eng.: English
EQ-5D-3L: EuroQuol – 5 Dimension – 3 Level Questionnaire
Fig.: Figure
Ger.: German
ICARS: International Cooperative Ataxia Rating Scale
ID: Identity
INAS: Inventory of Non-Ataxia Signs
MCI: Mild cognitive impairment
MMSE: Mini-Mental State Examination
MoCA: Montreal Cognitive Assessment
MSA-C: Multisystem Atrophy Cerebellar Type
PHQ-9: Patient Health Questionnaire (Depression Part)
ROCFT: Rey–Osterrieth Complex Figure Test
SARA: Scale for the Assessment and Rating of Ataxia
SCAFI: SpinoCerebellar Ataxia Functional Index
SAOA: Sporadic Adult Onset Ataxia
SCA1/2/3/6/8/14: Spinocerebellar Ataxia Type 1/2/3/6/8/14
Tab.: Table
UMEA: University Medicine Essen Clinician Scientist Academy
US (−American): United States (American)
vs.: Versus
WAIS-IV: Wechsler Adult Intelligence Scale – 4th edition
yrs.: Years
WMS-IV: Wechsler Memory Scale – 4th edition
