# Supplementary material for: Validation of a German version of the Cerebellar Cognitive Affective/ Schmahmann Syndrome Scale: preliminary version and study protocol
Source: Neurol Res Pract. 2020 Sep 29;2:39. doi: 10.1186/s42466-020-00071-3 (PMC7650062; doi:10.1186/s42466-020-00071-3)
Supplement: Supplementary file 1 — Additional file 1. [file 42466_2020_71_MOESM1_ESM.docx]

**Supplementary Materials**

1. **Introduction to the test items of the original CCAS Scale**
2. **Modifications of the German versions of the CCAS Scale resulting from the translation process and pretesting**
3. **Preliminary German versions B-D of the CCAS Scale**
4. **Detailed instructions of the American English CCAS Scale translated to German**
5. **References**
6. **Introduction to the test items of the original CCAS Scale**

The original American English version of the CCAS Scale contains twelve items. The following section describes the items and what cognitive domains they measure:

1. *Semantic Fluency*

The participant is asked to name as many *„animals/ living creatures“* as possible in one minute. This task recruits various cognitive domains such as attention, working memory (not repeating the same words within one minute), cognitive flexibility, problem solving (for generating search strategies), higher linguistic functions such as word processing and semantic memory [1-3].

1. *Phonemic Fluency*

In the phonemic fluency task the participant is required to name as many words that start with the letter *„F“* as possible in one minute. The same cognitive domains are tested as in the foregoing task.

1. *Category Switching*

Here, the participant is asked to alternate between the two categories *“professions/ jobs”* and *“vegetables”* and perform as many correct switches between these categories as possible in one minute. This item measures semantic memory, cognitive flexibility, coordination of retrieval processes and the ability for spontaneous word production as subdomains of executive functions [4].

1. *Verbal Registration*

Verbal short-term memory is tested using the verbal registration and delayed verbal recall task (see below) [1, 3]. In the verbal registration part the participant is required to memorize five words. According to Hoche and collaborators [1] inability of learning the five words after four attempts would be a sign for cerebral involvement (“red flag”). Note, that this item is not rated with a score and cannot be failed.

1. *Digit Span Forward*

Short-term memory (where information can be hold for seconds) and attention is examined using the digit span forward task [1, 3, 5]. Here, the participant is asked to repeat a sequence of numbers with an increasing length after the examiner has read them aloud once.

1. *Digit Span Backward*

Sequences of numbers are read aloud by the examiner with an increasing length. The participant is asked to repeat them backwards. This task measures attention and working memory (where information can be processed in contrast to short-term memory) [1, 5].

1. *Cube Draw*

To test visuospatial cognitive abilities, executive functions, i.e. problem solving, and metalinguistic abilities the cube draw task is applied [3, 6, 7]. In this task the participant is asked to draw a three-dimensional, transparent cube.

1. *Cube Copy*

If the foregoing cube draw task cannot be done accurately due to cognitive deficits (not due to motor deficits) the cube copy task is applied. Here, the participant is required to copy a three-dimensional, transparent cube. It captures visuospatial cognitive abilities just like the cube draw task but not metalinguistic abilities [1, 6, 8]. The cube draw and cube copy tasks are rated together and comprise one pass/ fail criterion (see Fig. 1 in main manuscript).

1. *Verbal Recall*

In this task the participant is asked to recall the five words that had to be memorized earlier in the verbal registration task (see above). If this is not possible the examiner gives standardized hints. If recall still is not possible, multiple-choice alternatives are given and the participant has to choose the right answer from these alternatives. If the participant cannot recall two or more words from the multiple-choice alternatives this raises concern about cerebral involvement because severe declarative memory loss is not typical for CCAS (“red flag”). This item is rated and used for calculation of total number of failed test items and total sum score [1].

1. *Similarities*

In this test item the participant is asked to name similarities of two words, i.e. what these two words have in common. For example *“the moon”* and *“a ball”* are both round. This task captures abilities in abstract reasoning [1, 5, 8].

1. *Go/No-go*

In the go/ no-go task the participant is required to lift one finger if the examiner knocks on the table once whereas he shall do nothing if the examiner knocks on the table twice. This test investigates visual attention and behavioral inhibition [1, 8].

1. *Affect*

In this section affective/ neuropsychiatric abnormalities are captured semi-quantitatively. The examiner can check from a given list of affective/ psychiatric abnormalities if they are present in the tested individual. This item is derived from another psychometric tool developed by Schmahmann`s group, namely the *Cerebellar Neuropsychiatric Rating Scale (CNRS)*. The CNRS captures the five affective/ neuropsychiatric core domains found to be pathological in patients with a CCAS: *attentional control, emotional control, autism-spectrum disorders, psychosis-spectrum disorders* and *social skills* [9].

Test Result Evaluation

Depending on reaching the specific cut-off value of a single test item, each single test item is rated as passed or failed. If only one item is failed a CCAS is possible, if two are failed a CCAS is probable, and if three or more are failed it is definite according to Hoche and collaborators [1]. The scores of the single test items can further be added to a sum score which is not used to diagnose a CCAS. Instead, it is intended to be used for comparison of individual performance in follow-up examinations. The minimum of total sum score is 0, the maximum is 120 (for details see [1] and Fig. 1). Of note, validation of the cut-off values and pass/ fail criteria of the German CCAS Scale in a cohort of German-speaking cerebellar patients is pending.

1. Modifications in the German versions of the CCAS Scale resulting from the translation process and pretesting

The test items of the original CCAS Scale have been translated and adapted to the specifics of the German language if needed. The resulting preliminary German version A of the CCAS Scale is shown in Fig. 1 in the main manuscript. Versions B-D are shown below in Supplements, Part 3.

1. *Semantic Fluency*

In the German version of the scale the instructions for the semantic fluency task have been translated (original American English = Eng.: “Please name as many animals or living creatures as you can in one minute.”; German = Ger.: “Bitte nennen Sie so viele Tiere oder Lebewesen wie Ihnen innerhalb einer Minute einfallen.”). No modifications have been made considering the semantic category (e.g. *“animals/ living creatures”* in version A) because no language- or culture-specific differences were to be expected.

1. *Phonemic Fluency*

In contrast to the semantic fluency task, language specific differences were to be expected in the phonemic fluency task because frequencies of words that start with a certain letter in German and (American) English might differ. Exact overall (i.e. not differentiating between verbs, nouns, names etc.) letter frequencies could not be found in the literature. To bypass this bias we decided to use letters in our German scales that are part of validated German cognitive tests, for example the German versions of the MoCA [10]. The instructions have been translated as follows: Eng.: “Please name as many words as you can in one minute that start with the letter F. Do not use names of people or places or repeat the same word in different forms.”; Ger.: “Bitte nennen Sie so viele Wörter wie möglich mit dem Anfangsbuchstaben „F”, wie Ihnen innerhalb einer Minute einfallen. Benutzen Sie nicht Namen von Personen oder Orten und wiederholen Sie nicht dasselbe Wort in unterschiedlichen Formen.“

In the subsequent pretesting experiment six examiners (2 psychologists and 4 neurologists) asked for a specification of the wording *“different forms”* (Ger.: “unterschiedliche Formen”). We decided to add to the verbal instruction that no different grammatical forms of the same word should be given. An example was added for clarification (see Fig. 1 in the main manuscript).

1. *Category Switching*

The same semantic categories have been chosen as in the original version since no language-specific differences were to be expected. Thus, the categories (e.g. *“profession/ job”* and *“vegetables”* for version A) were simply translated (Eng.: “Please name a type of vegetable and then a type of profession or job, and then another vegetable and another profession, and so on, switching between the two lists. Name as many as you can in one minute.”; Ger.: “Bitte nennen Sie eine Gemüsesorte und dann einen Beruf oder einen Job, dann eine andere Gemüsesorte und dann wieder einen weiteren Beruf usw. Wechseln Sie immer zwischen den beiden Kategorien ab. Nennen Sie so viele Wörter wie Ihnen innerhalb einer Minute einfallen.“).

1. *Verbal Registration*

Again, the main part of the instructions has been translated without further change (Eng.: “I am going to read you a list of words which I would like you to learn. Please repeat these words. I am going to ask you to give them back in a few minutes. (*Read 5 words at rate of 1/ second. Subject repeats them once, then repeats them again. Repeat trials until subject recalls all 5 words. Stop after 4 attempts.) Flower – Robert – Courage – Speak – Yellow* ”; Ger.: “Ich werde Ihnen nun eine Wortliste vorlesen, welche Sie bitte lernen sollen. Bitte wiederholen und merken Sie sich die Wörter. Ich werde Sie in einigen Minuten noch einmal bitten, diese Wörter wiederzugeben. *(Lesen Sie die 5 Wörter in einer Geschwindigkeit von 1/ Sekunde vor. Die Testperson wiederholt die Wörter einmal, dann wiederholt sie diese ein weiteres Mal. Wiederholen Sie die Wortliste bis die Testperson alle 5 Wörter erinnert. Brechen Sie nach 4 Versuchen ab.*) Blume – Frank – Mut – sprechen – gelb“). Only the names were replaced, e.g. *“Robert”* in version A was replaced by *“Frank”* because *“Frank”* is a similarly common male name in Germany as *“Robert”* is in the United States.

During the pretesting phase two questions arose. Firstly, it was unclear whether the participant should repeat the words twice instantly after the examiner has once read them aloud, or whether the examiner reads the words aloud a first time, then lets the participant repeat them, before reading them again and letting the participant repeat them a second time. This concern was raised by one neurologist in pretesting. Secondly, it was unclear if the order in which the words are repeated plays a role. One neurologist and two neuropsychologists expressed their concern.

These concerns were solved after consultation with the senior author of the original US American CCAS Scale (J.D. Schmahmann). Firstly, after the examiner has read the words aloud once the participant repeats them. Then the participant repeats them a second time with the examiner’s prompt. This procedure may be repeated until the participant recalls all five words, but it is stopped after four attempts. Secondly, the participant may repeat the words in any order, although the original authors have noted that lack of self-organization/ self-monitoring of the response may be an indicator of impaired strategy formation in the learning process. The detailed test instructions have also been revised accordingly.

1. *Digit Span Forward*

Digit sequences have been copied from the American English original. Here, they have been generated using a randomization software [1]. Other than translation no modifications have been made (Eng.: “I am going to read you some numbers. Please repeat them in exactly the same order. *(Read aloud at a rate of 1 per second. Start with * and administer previous items if subject fails to repeat *)*”; Ger.: “Ich werde Ihnen nun einige Zahlen jeweils einmal vorlesen. Bitte wiederholen Sie diese in der exakt gleichen Reihenfolge. *(Lesen Sie die Zahlen in einer Geschwindigkeit von 1/ Sekunde laut vor. Beginnen Sie beim * und führen Sie die vorherigen Zahlenspannen nur durch, wenn * falsch wiedergegeben wurde)*“).

1. *Digit Span Backward*

Again, the digit sequences have been copied from the American English original [1]. Other than translation no modifications have been made (Eng.: “Now please say these numbers backwards, in reverse order. *(Give example, then start with *)*”; Ger.: “Nun geben Sie die Zahlen bitte rückwärts wieder, also in umgekehrter Reihenfolge. *(Machen Sie das Beispiel vor, dann beginnen Sie beim*)*“).

1. *Cube Draw*

In the cube draw task, the translation had to be adapted to German language (Eng.: “Please draw a cube – a six-sided box, make it transparent or see-through. *(Use space bottom left)*”; Ger.: “Bitte zeichnen Sie einen Würfel – einen sechsseitigen Kasten. Zeichnen Sie ihn transparent, also durchsichtig. *(Nutzen Sie den unten links)*”). The German word for *“box”* *(“Kasten”)* was ambiguous (issue raised by two patients and one neurologist) in our prototype translation of the instructions used for pretesting. Therefore, it was rejected. The word *“transparent”* needed further specification as well (issue raised by one patient and one neurologist). Because we found this word to be quite important for the correct understanding of the instructions it was kept, and we decided that further explanation by the experimenter is allowed if needed (for the preliminary version of the instructions see Fig. 1 in main manuscript).

1. *Cube Copy*

The instructions for the cube copy task were translated (Eng.: “Please copy the cube shown on page 2. *(Neatness not scored)*”; Ger.: “Bitte zeichnen Sie den Würfel ab, der auf Seite 2 abgebildet ist. *(Sorgfalt/ Genauigkeit wird nicht bewertet)*“). No further changes were made.

1. *Verbal Recall*

The instructions for the verbal recall task as well as cues and multiple-choice alternatives given at the end of the original CCAS Scale were translated (Eng.: “What were the words I asked you to learn earlier? *(Subjects recalls the words learned previously. Use cues and multiple-choice alternatives bottom left if needed)*”; Ger.: “Wie lauten die Wörter, die ich Sie vor einer Weile gebeten habe, zu lernen? *(Die Testperson gibt die Wörter, die sie sich vorher merken sollte, wieder. Benutzen Sie die Hinweise und die Mehrfachauswahl (unten), wenn notwendig.)”*). No further modifications were made.

1. *Similarities*

Like the semantic fluency and category switching tasks the semantic categories were kept the same in the German CCAS Scale versions because no language-specific differences were to be expected (Eng.: “How are the following words alike; what is the same about them? *(Provide example, then test items.)*”; Ger.: “Was haben die folgenden Wörter gemeinsam? Was ist ihre Gemeinsamkeit? *(Geben Sie ein Beispiel, testen Sie danach die Items.)*“). No further change was necessary.

1. *Go/No-go*

Since this test should not be affected by language the directions were solely translated (Eng.: “I am going to tap the table. When I tap once, please raise your finger, then put it back down again. When I tap twice, don`t do anything. *(Give an example of each condition to make sure subject understands)*”; Ger.: “Ich werde nun auf den Tisch klopfen. Wenn ich einmal klopfe, heben Sie bitte den Finger und senken Sie ihn wieder. Wenn ich zweimal klopfe, machen Sie bitte nichts. *(Geben Sie ein Beispiel für jede Bedingung, um sicherzugehen, dass die Testperson die Anweisung verstanden hat)*“). The sequence of binary test conditions (go = condition 1/ no-go = condition 2) were copied from the original scale.

1. *Affect*

No modifications were made. Only translation was performed (Eng.: *“Rater assesses if the following are present, incorporating input from patient and/ or caregiver.*”; Ger.: *“Der Testleiter schätzt ein, ob die folgenden Auffälligkeiten* *vorliegen, Eindrücke von der Testperson oder deren engen Bezugspersonen werden mit einbezogen.*“). See Fig. 1 in main manuscript.

*Total Sum Score and Number of Failed Tests*

No modifications were made. Only translation was performed (Eng.: *“Calculate total raw score (1^st^ column) and total number of failed tests (2^nd^ column). 1 failed test = possible CCAS*; 2 failed tests = probable CCAS; 3 or more failed tests = definite CCAS”; Ger.: „*Berechnen Sie den Gesamtwert (1. Spalte) und den Gesamtwert der „Nicht-Bestandenen“ Aufgaben (2. Spalte). 1 „Nicht-Bestandene“ Aufgabe = mögliches CCAS; 2 „Nicht-Bestandene“ Aufgaben = wahrscheinliches CCAS; 3* oder mehr *„Nicht-Bestandene*” Aufgaben = definitives CCAS“). Again, please note that validation of the original cut-off values and pass/ fail criteria in a cohort of German-speaking cerebellar patients is pending.

*Layout*

The layout of the German scale differs slightly from the American English original. During pretesting several examiners and participants indicated that they were able to see the five words from the verbal registration task when drawing the cube in the cube draw task onto the bottom of page 1 of the CCAS Scale. Furthermore, the space for cube drawing was very limited considering the severe cerebellar motor syndrome in some patients. Therefore, space for the cube draw task was provided on a second (otherwise empty) page. A similar modification was made for the cube copy task. Space was provided on the fourth page necessary to prevent the participant from seeing the cube in the cube draw task.

3. Preliminary German versions B-D of the CCAS Scale (on the following pages)

**[Note: The German CCAS Scale has not been validated yet, and especially the cut-off values may change.]**

**CEREBELLAR COGNITIVE AFFECTIVE /
SCHMAHMANN SYNDROME Skala (CCAS-Skala) ID#: Bildungsjahre:
VERSION 1B. Datum:**

| **Semantische Wortflüssigkeit** | | | | | | | **Punkte = alle korrekten Wörter (maximal 26 Wörter). „Nicht-Bestanden“ bei 15 Punkten oder weniger.**  *(Nutzen Sie den Platz unten für Notizen.)* | | | | | | | | | | | | | | | | | | | | | | | | | | | **Punkte** | **Bestanden= 0**  **Nicht-Bestanden= 1** |
| --- | --- | --- | --- | --- | --- | --- | --- | --- | --- | --- | --- | --- | --- | --- | --- | --- | --- | --- | --- | --- | --- | --- | --- | --- | --- | --- | --- | --- | --- | --- | --- | --- | --- | --- | --- |
| Bitte nennen Sie so viele Kleidungsstücke wie Ihnen innerhalb einer Minute einfallen. | | | | | | | | | | | | | | | | | | | | | | | | | | | | | | | | | | **/26** |  |
| **Phonematische Wortflüssigkeit** | | | | | | | **Punkte = alle korrekten Wörter (maximal 19 Wörter). „Nicht-Bestanden“ bei 9 Punkten oder weniger.**  *(Nutzen Sie den Platz unten für Notizen.)* | | | | | | | | | | | | | | | | | | | | | | | | | | | **/19** |  |
| Bitte nennen Sie so viele Wörter wie möglich mit dem Anfangsbuchstaben *„S“* wie Ihnen innerhalb einer Minute einfallen. Benutzen Sie nicht Namen von Personen oder Orten und wiederholen Sie nicht dasselbe Wort in unterschiedlichen Formen. *(Unterschiedliche Formen desselben Wortes sind z.B. „der Fisch, des Fisches, die Fische“)* | | | | | | | | | | | | | | | | | | | | | | | | | | | | | | | | | |  |  |
| **Kategorie-Wechsel** | | | | | | | **Punkte = Anzahl der korrekten Wechsel zwischen den Wortkategorien (maximal 15 Wechsel). Wiederholungen und Regelbrüche werden nicht bewertet. „Nicht-Bestanden“ bei 9 Punkten oder weniger.** *(Nutzen Sie den Platz unten für Notizen.)* | | | | | | | | | | | | | | | | | | | | | | | | | | | **/15** |  |
| Bitte nennen Sie eine Obstsorte und dann eine Stadt, dann eine andere Obstsorte und dann wieder eine weitere Stadt usw. Wechseln Sie immer zwischen den beiden Kategorien ab. Nennen Sie so viele Wörter wie Ihnen innerhalb einer Minute einfallen. | | | | | | | | | | | | | | | | | | | | | | | | | | | | | | | | | |  |  |
| **Verbales Lernen** | | | | | | | **Diese Aufgabe wird nicht mit Punkten bewertet. (Wenn 4 Versuche benötigt werden, die 5 Wörter zu lernen, ist dies ein Hinweis für eine zerebrale Beteiligung.)** | | | | | | | | | | | | | | | | | | | | | | | | | | |  |  |
| Ich werde Ihnen nun eine Wortliste vorlesen, welche Sie bitte lernen sollen. Bitte wiederholen und merken Sie sich die Wörter. Ich werde Sie in einigen Minuten noch einmal bitten, diese Wörter wiederzugeben.  *(Lesen Sie die 5 Wörter in einer Geschwindigkeit von 1/Sekunde vor. Die Testperson wiederholt die Wörter einmal, dann wiederholt sie diese ein weiteres Mal. Wiederholen Sie die Wortliste bis die Testperson alle 5 Wörter erinnert. Brechen Sie nach 4 Versuchen ab.)* | | | | | | | | | | | | | | | | | | | | | | | | | | | | | | | | | |  |  |
|  | | | [Schnee] | | | | | | [Bus] | | | | | | | [Schicksal] | | | | | | | [rennen] | | | | | | | [groß] | | | |  |  |
| Versuch 1 | | | [ ] | | | | | | [ ] | | | | | | | [ ] | | | | | | | [ ] | | | | | | | [ ] | | | |  |  |
| Versuch 2 | | | [ ] | | | | | | [ ] | | | | | | | [ ] | | | | | | | [ ] | | | | | | | [ ] | | | |  |  |
| Versuch 3 | | | [ ] | | | | | | [ ] | | | | | | | [ ] | | | | | | | [ ] | | | | | | | [ ] | | | |  |  |
| Versuch 4 | | | [ ] | | | | | | [ ] | | | | | | | [ ] | | | | | | | [ ] | | | | | | | [ ] | | | |  |  |
| **Zahlenspanne Vorwärts** | | | | | | | **Punkte = längste korrekt wiederholte Zahlenspanne. „Nicht-Bestanden“ bei 5 Punkten oder weniger.** | | | | | | | | | | | | | | | | | | | | | | | | | | | **/8** |  |
| Ich werde Ihnen nun einige Zahlen jeweils einmal vorlesen. Bitte wiederholen Sie diese in der exakt gleichen Reihenfolge. *(Lesen Sie die Zahlen in einer Geschwindigkeit von 1/Sekunde laut vor. Beginnen Sie beim * und führen Sie die vorherigen Zahlenspannen nur durch, wenn * falsch wiedergegeben wurde.)* | | | | | | | | | | | | | | | | | | | | | | | | | | | | | | | | | |  |  |
| 9-1 | [ ] | | | 0-4-8-6* | | | | | | [ ] | | | | | 2-8-3-6-1-4 | | | | | [ ] | | | | | 1-4-2-3-9-0-6-8 | | | | | | | [ ] | |  |  |
| 5-2-7 | [ ] | | | 3-5-9-7-0 | | | | | | [ ] | | | | | 8-0-7-5-9-6-3 | | | | | [ ] | | | | |  | | | | | | |  | |  |  |
| **Zahlenspanne Rückwärts** | | | | | | | **Punkte = längste korrekt wiedergegebene Zahlenspanne. „Nicht-Bestanden“ bei 3 Punkten oder weniger. Können 2 Ziffern nicht korrekt rückwärts wiedergegeben werden, werden 0 Punkte vergeben.** | | | | | | | | | | | | | | | | | | | | | | | | | | | **/6** |  |
| Nun geben Sie die Zahlen bitte rückwärts wieder, also in umgekehrter Reihenfolge. *(Machen Sie das Beispiel vor, dann beginnen Sie beim *.)* | | | | | | | | | | | | | | | | | | | | | | | | | | | | | | | | | |  |  |
| Bsp.: 5-8 = 8-5 | | *5-0 | | | | [ ] | | 2-9-7 | | | | [ ] | | 4-8-3-1 | | | | | [ ] | | 6-9-0-4-8 | | | | | [ ] | 5-3-2-1-7-0 | | | | | | [ ] |  |  |
| **Würfel (Zeichnen)** | | | | | | | **Punkte = 15 Punkte, wenn 12 Linien gezeichnet wurden und die Zeichnung dreidimensional ist. Hat die Zeichnung nicht 12 Linien oder ist nicht dreidimensional, führen Sie die Aufgabe *„Würfel (Abzeichnen)“* durch.** | | | | | | | | | | | | | | | | | | | | | | | | | | | **/15** |  |
| Bitte zeichnen Sie einen sechsseitigen Würfel. Zeichnen Sie ihn transparent, also durchsichtig. *(Nutzen Sie den Platz auf Seite 2.)* | | | | | | | | | | | | | | | | | | | | | | | | | | | | | | | | | |  |  |
| **Würfel (Abzeichnen)** | | | | | | | **Punkte = 12 Punkte, 1 Punkt pro Linie. Ziehen Sie jeweils 1 Punkt ab, wenn die Zeichnung nicht dreidimensional ist, 1 Punkt für jede Linie, die nicht gezeichnet wurde und 1 Punkt für jede zusätzlich gezeichnete Linie > 12. „Nicht-Bestanden“ bei 11 Punkten oder weniger.** | | | | | | | | | | | | | | | | | | | | | | | | | | |  |  |
| Bitte zeichnen Sie den Würfel ab, der auf Seite 4 abgebildet ist. *(Sorgfalt/Genauigkeit wird nicht bewertet.)* | | | | | | | | | | | | | | | | | | | | | | | | | | | | | | | | | |  |  |
|  | | | | | | | | | | | | | | | | | | *Notizen:* | | | | | | | | | | | | | | | | | |
| Semantische WF | | | | | | | | | | | | | Phonematische WF | | | | | | | | | | | | | | | Kategorie-Wechsel | | | | | | | |
|  | | | | | | |  | | | | | | | | | | | | | | | | | | | | | | | | | | |  |  |
| **[Note: The German CCAS Scale has not been validated yet, and especially the cut-off values may change.]**  *Zeichnen Sie den Würfel hier.* | | | | | | | | | | | | | | | | | | | | | | | | | | | | | | | | | | | |
| **[Note: The German CCAS Scale has not been validated yet, and especially the cut-off values may change.]** | | | | | | | | | | | | | | | | | | | | | | | | | | | | | | | | | | | |
| **Verbaler Abruf** | | | | | | | **Spontan = 3 Punkte pro Wort, Kategorie = 2 Punkte, Mehrfachauswahl = 1 Punkt. Punkte = Gesamtpunktzahl. „Nicht-Bestanden“ bei 10 Punkten oder weniger. Wird nicht mehr als ein Wort bei der Mehrfachauswahl erinnert, ist dies ein Hinweis für eine zerebrale Beteiligung.** | | | | | | | | | | | | | | | | | | | | | | | | | | | **Punkte** | **Bestanden= 0**  **Nicht-Bestanden= 1** |
| Wie lauten die Wörter, die ich Sie vor einer Weile gebeten habe, zu lernen? *(Die Testperson gibt die Wörter, die sie sich vorher merken sollte, wieder. Benutzen Sie die Hinweise und die Mehrfachauswahl (unten), wenn notwendig.)* | | | | | | | | | | | | | | | | | | | | | | | | | | | | | | | | | | **/15** |  |
|  | | | | | [Schnee] | | | | | | [Bus] | | | | | | [Schicksal] | | | | | | | [rennen] | | | | | | | [groß] | | |  |  |
| Spontane Wiedergabe | | | | | [ ] | | | | | | [ ] | | | | | | [ ] | | | | | | | [ ] | | | | | | | [ ] | | |  |  |
| Mit Hinweis | | | | | [ ] | | | | | | [ ] | | | | | | [ ] | | | | | | | [ ] | | | | | | | [ ] | | |  |  |
| Mit Mehrfachauswahl | | | | | [ ] | | | | | | [ ] | | | | | | [ ] | | | | | | | [ ] | | | | | | | [ ] | | |  |  |
| **Gemeinsamkeiten** | | | | | | | **Korrekte Antwort (konzeptuell) = 2 Punkte, teilweise korrekte Antwort (konkret) = 1 Punkt, inkorrekte/ keine Antwort = 0 Punkte. Punkte = Gesamtpunktzahl. „Nicht-Bestanden“ bei 6 Punkten oder weniger. Auswertungshilfe – unten und in der Durchführungsanleitung.** | | | | | | | | | | | | | | | | | | | | | | | | | | | **/8** |  |
| Was haben die folgenden Wörter gemeinsam? Was ist ihre Gemeinsamkeit? *(Geben Sie ein Beispiel, testen Sie danach die Items.)* | | | | | | | | | | | | | | | | | | | | | | | | | | | | | | | | | |  |  |
| Bsp.: Schaf/Elefant = Tiere | | | | | 1.argwöhnisch/eifersüchtig | | | | | | | | | 2.Würfel/Dreieck | | | | | | | | 3.Stuhl/Tisch | | | | | | | 4.Wolle/Seide | | | | |  |  |
|  |  |  |  |  | [ /2] | | | | | | | | | [ /2] | | | | | | | | [ /2] | | | | | | | [ /2] | | | | |  |  |
| **Go/No-Go** | | | | | | | **2 Punkte für keinen Fehler, 1 Punkt für 1 Fehler, 0 Punkte für 2 oder mehr Fehler. Punkte = Gesamtpunktzahl. „Nicht-Bestanden“ bei 0 Punkten.** | | | | | | | | | | | | | | | | | | | | | | | | | | | **/2** |  |
| Ich werde nun auf den Tisch klopfen. Wenn ich einmal klopfe, heben Sie bitte den Finger und senken Sie ihn wieder! Wenn ich zweimal klopfe, machen Sie bitte nichts! *(Geben Sie ein Beispiel für jede Bedingung, um sicherzugehen, dass die Testperson die Anweisung verstanden hat.)*  **2 – 1 – 1 – 2 – 1 – 2 – 1 – 2 – 2 – 1 – 1 – 2 – 1 – 2** | | | | | | | | | | | | | | | | | | | | | | | | | | | | | | | | | |  |  |
| **Affekt** | | | | | | | **Geben Sie 6 Punkte, wenn keine Auffälligkeiten vorliegen. Ziehen Sie 1 Punkt pro vorhandenes Item ab. „Nicht-Bestanden“ bei 4 Punkten oder weniger.**  *(Der Testleiter schätzt ein, ob die folgenden Auffälligkeiten vorliegen, Eindrücke von der Testperson oder deren engen Bezugspersonen werden miteinbezogen.)* | | | | | | | | | | | | | | | | | | | | | | | | | | | **/6** |  |
| [ ] Hat Schwierigkeiten die Aufmerksamkeit zu fokussieren oder bei der mentalen Flexibilität  [ ] Emotional labil, unpassende/inkongruente Emotionen, erscheint hoffnungslos oder depressiv  [ ] Zeigt schnell Zeichen einer Reizüberflutung oder ausweichendes Verhalten  [ ] Äußert unlogische Gedanken oder Paranoia  [ ] Zeigt fehlende Empathie, ist apathisch oder zeigt eine Affektverarmung  [ ] Ist zornig oder aggressiv, reizbar, konfrontativ, hat Schwierigkeiten mit sozialen Grenzen oder sozialen  Regeln/Normen | | | | | | | | | | | | | | | | | | | | | | | | | | | | | | | | | |  |  |
| **GESAMTWERT** | | | | | | | | | | | | | | | | | | | | | | | | | | | | | | | | | | **/120** | **/10** |
| **Berechnen Sie den Gesamtwert (1. Spalte) und den Gesamtwert der „Nicht-Bestandenen“ Aufgaben (2. Spalte).**  **1 „Nicht-Bestandene“ Aufgabe = mögliches CCAS; 2 „Nicht-Bestandene“ Aufgaben = wahrscheinliches CCAS; 3 oder mehr „Nicht-Bestandene“ Aufgaben = definitives CCAS.** | | | | | | | | | | | | | | | | | | | | | | | | | | | | | | | | | | | |

| Hinweise und Mehrfachauswahl für die Aufgabe „Verbaler Abruf“ | | | | | |
| --- | --- | --- | --- | --- | --- |
| **Testwort** | **Schnee** | **Bus** | **Schicksal** | **rennen** | **groß** |
| **Hinweis** | Niederschlagsform | Verkehrsmittel, das Personen/Dinge befördert | Zukunftskonzept | etwas, das wir mit unseren Beinen tun | beschreibt die Größe eines Objekts |
| **Mehrfach- auswahl** | Regen | Lastwagen | Absicht | rennen | klein |
|  | Graupel | Zug | Schicksal | gehen | umfangreich |
|  | Schnee | Schiff | Vorhersage | springen | winzig |
|  | Hagel | Bus | Erwartung | hüpfen | groß |

| **Gemeinsamkeiten** | **Korrekte konzeptuelle Antwort (Beispiele)** | **Teilweise korrekte/konkrete Antwort (Beispiele)** |
| --- | --- | --- |
| argwöhnisch/eifersüchtig | (negative) Emotion | macht traurig/wütend/verärgert |
| Würfel/Dreieck | geometrische Form | man zeichnet sie |
| Stuhl/Tisch | Möbelstück | man stellt Dinge darauf, haben Beine |
| Wolle/Seide | Stoff, Material, kommt von Tieren | man trägt sie, sind weich |

**[Note: The German CCAS Scale has not been validated yet, and especially the cut-off values may change.]**

*Zeichnen Sie den Würfel hierhin ab.*

**Hoche, Guell, Vangel, Sherman, Schmahmann, Ataxia Center, Cognitive Behavioral Neurology Unit, Schmahmann Laboratory for Neuroanatomy and Cerebellar Neurobiology, Department of Neurology, Massachusetts General Hospital.**

**© 2016 The General Hospital Corporation. All Rights Reserved.**

**[Note: The German CCAS Scale has not been validated yet, and especially the cut-off values may change.]**

**CEREBELLAR COGNITIVE AFFECTIVE /
SCHMAHMANN SYNDROME Skala (CCAS-Skala) ID#: Bildungsjahre:
VERSION 1C. Datum:**

| **Semantische Wortflüssigkeit** | | | | | | | **Punkte = alle korrekten Wörter (maximal 26 Wörter). „Nicht-Bestanden“ bei 15 Punkten oder weniger.**  *(Nutzen Sie den Platz unten für Notizen.)* | | | | | | | | | | | | | | | | | | | | | | | | | | | | **Punkte** | **Bestanden= 0**  **Nicht-Bestanden= 1** |
| --- | --- | --- | --- | --- | --- | --- | --- | --- | --- | --- | --- | --- | --- | --- | --- | --- | --- | --- | --- | --- | --- | --- | --- | --- | --- | --- | --- | --- | --- | --- | --- | --- | --- | --- | --- | --- |
| Bitte nennen Sie so viele Sportarten wie Ihnen innerhalb einer Minute einfallen. | | | | | | | | | | | | | | | | | | | | | | | | | | | | | | | | | | | **/26** |  |
| **Phonematische Wortflüssigkeit** | | | | | | | **Punkte = alle korrekten Wörter (maximal 19 Wörter). „Nicht-Bestanden“ bei 9 Punkten oder weniger.**  *(Nutzen Sie den Platz unten für Notizen.)* | | | | | | | | | | | | | | | | | | | | | | | | | | | | **/19** |  |
| Bitte nennen Sie so viele Wörter wie möglich mit dem Anfangsbuchstaben *„P“* wie Ihnen innerhalb einer Minute einfallen. Benutzen Sie nicht Namen von Personen oder Orten und wiederholen Sie nicht dasselbe Wort in unterschiedlichen Formen. *(Unterschiedliche Formen desselben Wortes sind z.B. „der Fisch, des Fisches, die Fische“)* | | | | | | | | | | | | | | | | | | | | | | | | | | | | | | | | | | |  |  |
| **Kategorie-Wechsel** | | | | | | | **Punkte = Anzahl der korrekten Wechsel zwischen den Wortkategorien (maximal 15 Wechsel). Wiederholungen und Regelbrüche werden nicht bewertet. „Nicht-Bestanden“ bei 9 Punkten oder weniger.** *(Nutzen Sie den Platz unten für Notizen.)* | | | | | | | | | | | | | | | | | | | | | | | | | | | | **/15** |  |
| Bitte nennen Sie ein Instrument und dann ein Körperteil, dann ein anderes Instrument und dann wieder ein weiteres Körperteil usw. Wechseln Sie immer zwischen den beiden Kategorien ab. Nennen Sie so viele Wörter wie Ihnen innerhalb einer Minute einfallen. | | | | | | | | | | | | | | | | | | | | | | | | | | | | | | | | | | |  |  |
| **Verbales Lernen** | | | | | | | **Diese Aufgabe wird nicht mit Punkten bewertet. (Wenn 4 Versuche benötigt werden, die 5 Wörter zu lernen, ist dies ein Hinweis für eine zerebrale Beteiligung.)** | | | | | | | | | | | | | | | | | | | | | | | | | | | |  |  |
| Ich werde Ihnen nun eine Wortliste vorlesen, welche sie bitte lernen sollen. Bitte wiederholen und merken Sie sich die Wörter. Ich werde Sie in einigen Minuten noch einmal bitten, diese Wörter wiederzugeben.  *(Lesen Sie die 5 Wörter in einer Geschwindigkeit von 1/Sekunde vor. Die Testperson wiederholt die Wörter einmal, dann wiederholt sie diese ein weiteres Mal. Wiederholen Sie die Wortliste bis die Testperson alle 5 Wörter erinnert. Brechen Sie nach 4 Versuchen ab.)* | | | | | | | | | | | | | | | | | | | | | | | | | | | | | | | | | | |  |  |
|  | | [Dorf] | | | | | | | | | [Petra] | | | | | | | [Glück] | | | | | | [antworten] | | | | | | | [viereckig] | | | |  |  |
| Versuch 1 | | [ ] | | | | | | | | | [ ] | | | | | | | [ ] | | | | | | [ ] | | | | | | | [ ] | | | |  |  |
| Versuch 2 | | [ ] | | | | | | | | | [ ] | | | | | | | [ ] | | | | | | [ ] | | | | | | | [ ] | | | |  |  |
| Versuch 3 | | [ ] | | | | | | | | | [ ] | | | | | | | [ ] | | | | | | [ ] | | | | | | | [ ] | | | |  |  |
| Versuch 4 | | [ ] | | | | | | | | | [ ] | | | | | | | [ ] | | | | | | [ ] | | | | | | | [ ] | | | |  |  |
| **Zahlenspanne Vorwärts** | | | | | | | **Punkte = längste korrekt wiederholte Zahlenspanne. „Nicht-Bestanden“ bei 5 Punkten oder weniger.** | | | | | | | | | | | | | | | | | | | | | | | | | | | | **/8** |  |
| Ich werde Ihnen nun einige Zahlen jeweils einmal vorlesen. Bitte wiederholen Sie diese in der exakt gleichen Reihenfolge. *(Lesen Sie die Zahlen in einer Geschwindigkeit von 1/Sekunde laut vor. Beginnen Sie beim * und führen Sie die vorherigen Zahlenspannen nur durch, wenn * falsch wiedergegeben wurde.)* | | | | | | | | | | | | | | | | | | | | | | | | | | | | | | | | | | |  |  |
| 4-0 | [ ] | | | 2-8-3-7* | | | | | | [ ] | | | | | 0-2-5-4-6-3 | | | | | | [ ] | | | | | 1-0-8-3-7-4-6-2 | | | | | | | [ ] | |  |  |
| 6-1-5 | [ ] | | | 2-0-3-1-9 | | | | | | [ ] | | | | | 9-8-1-7-2-4-8 | | | | | | [ ] | | | | |  | | | | | | |  | |  |  |
| **Zahlenspanne Rückwärts** | | | | | | | **Punkte = längste korrekt wiedergegebene Zahlenspanne. „Nicht-Bestanden“ bei 3 Punkten oder weniger. Können 2 Ziffern nicht korrekt rückwärts wiedergegeben werden, werden 0 Punkte vergeben.** | | | | | | | | | | | | | | | | | | | | | | | | | | | | **/6** |  |
| Nun geben Sie die Zahlen bitte rückwärts wieder, also in umgekehrter Reihenfolge. *(Machen Sie das Beispiel vor, dann beginnen Sie beim *.)* | | | | | | | | | | | | | | | | | | | | | | | | | | | | | | | | | | |  |  |
| Bsp.: 5-8 = 8-5 | | | *3-2 | | | | | [ ] | 8-1-4 | | | | [ ] | | | | 0-7-6-9 | | | [ ] | | 3-5-2-0-6 | | | | | [ ] | 1-5-8-7-3-9 | | | | | | [ ] |  |  |
| **Würfel (Zeichnen)** | | | | | | | **Punkte = 15 Punkte, wenn 12 Linien gezeichnet wurden und die Zeichnung dreidimensional ist. Hat die Zeichnung nicht 12 Linien oder ist nicht dreidimensional, führen Sie die Aufgabe *„Würfel (Abzeichnen)“* durch.** | | | | | | | | | | | | | | | | | | | | | | | | | | | | **/15** |  |
| Bitte zeichnen Sie einen sechsseitigen Würfel. Zeichnen Sie ihn transparent, also durchsichtig. *(Nutzen Sie den Platz auf Seite 2.)* | | | | | | | | | | | | | | | | | | | | | | | | | | | | | | | | | | |  |  |
| **Würfel (Abzeichnen)** | | | | | | | **Punkte = 12 Punkte, 1 Punkt pro Linie. Ziehen Sie jeweils 1 Punkt ab, wenn die Zeichnung nicht dreidimensional ist, 1 Punkt für jede Linie, die nicht gezeichnet wurde und 1 Punkt für jede zusätzlich gezeichnete Linie > 12. „Nicht-Bestanden“ bei 11 Punkten oder weniger.** | | | | | | | | | | | | | | | | | | | | | | | | | | | |  |  |
| Bitte zeichnen Sie den Würfel ab, der auf Seite 4 abgebildet ist. *(Sorgfalt/Genauigkeit wird nicht bewertet.)* | | | | | | | | | | | | | | | | | | | | | | | | | | | | | | | | | | |  |  |
|  | | | | | | | | | | | | | | | | | | *Notizen:* | | | | | | | | | | | | | | | | | | |
| Semantische WF | | | | | | | | | | | | | | Phonematische WF | | | | | | | | | | | | | | | | Kategorie-Wechsel | | | | | | |
|  | | | | | |  | | | | | | | | | | | | | | | | | | | | | | | | | | | | |  |  |
| **[Note: The German CCAS Scale has not been validated yet, and especially the cut-off values may change.]**  *Zeichnen Sie den Würfel hier.*  **[Note: The German CCAS Scale has not been validated yet, and especially the cut-off values may change.]** | | | | | | | | | | | | | | | | | | | | | | | | | | | | | | | | | | | | |
| **Verbaler Abruf** | | | | | | **Spontan = 3 Punkte pro Wort, Kategorie = 2 Punkte, Mehrfachauswahl = 1 Punkt. Punkte = Gesamtpunktzahl. „Nicht-Bestanden“ bei 10 Punkten oder weniger. Wird nicht mehr als ein Wort bei der Mehrfachauswahl erinnert, ist dies ein Hinweis für eine zerebrale Beteiligung.** | | | | | | | | | | | | | | | | | | | | | | | | | | | | | **Punkte** | **Bestanden= 0**  **Nicht-Bestanden= 1** |
| Wie lauten die Wörter, die ich Sie vor einer Weile gebeten habe, zu lernen? *(Die Testperson gibt die Wörter, die sie sich vorher merken sollte, wieder. Benutzen Sie die Hinweise und die Mehrfachauswahl (unten), wenn notwendig.)* | | | | | | | | | | | | | | | | | | | | | | | | | | | | | | | | | | | **/15** |  |
|  | | | | | [Dorf] | | | | | | | [Petra] | | | | | | | [Glück] | | | | | | [antworten] | | | | | | | [viereckig] | | |  |  |
| Spontane Wiedergabe | | | | | [ ] | | | | | | | [ ] | | | | | | | [ ] | | | | | | [ ] | | | | | | | [ ] | | |  |  |
| Mit Hinweis | | | | | [ ] | | | | | | | [ ] | | | | | | | [ ] | | | | | | [ ] | | | | | | | [ ] | | |  |  |
| Mit Mehrfachauswahl | | | | | [ ] | | | | | | | [ ] | | | | | | | [ ] | | | | | | [ ] | | | | | | | [ ] | | |  |  |
| **Gemeinsamkeiten** | | | | | | **Korrekte Antwort (konzeptuell) = 2 Punkte, teilweise korrekte Antwort (konkret) = 1 Punkt, inkorrekte/ keine Antwort = 0 Punkte. Punkte = Gesamtpunktzahl. „Nicht-Bestanden“ bei 6 Punkten oder weniger. Auswertungshilfe – unten und in der Durchführungsanleitung.** | | | | | | | | | | | | | | | | | | | | | | | | | | | | | **/8** |  |
| Was haben die folgenden Wörter gemeinsam? Was ist ihre Gemeinsamkeit? *(Geben Sie ein Beispiel, testen Sie danach die Items.)* | | | | | | | | | | | | | | | | | | | | | | | | | | | | | | | | | | |  |  |
| Bsp.: Schaf/Elefant = Tiere | | | | | | 1.Orange/Karotte | | | | | | | | | | 2.Schnecke/Krabbe | | | | | | | 3.Schuhe/Gürtel | | | | | | 4.Buch/Zeitung | | | | | |  |  |
|  |  |  |  |  |  | [ /2] | | | | | | | | | | [ /2] | | | | | | | [ /2] | | | | | | [ /2] | | | | | |  |  |
| **Go/No-Go** | | | | | | **2 Punkte für keinen Fehler, 1 Punkt für 1 Fehler, 0 Punkte für 2 oder mehr Fehler. Punkte = Gesamtpunktzahl. „Nicht-Bestanden“ bei 0 Punkten.** | | | | | | | | | | | | | | | | | | | | | | | | | | | | | **/2** |  |
| Ich werde nun auf den Tisch klopfen. Wenn ich einmal klopfe, heben Sie bitte den Finger und senken Sie ihn wieder! Wenn ich zweimal klopfe, machen Sie bitte nichts! *(Geben Sie ein Beispiel für jede Bedingung, um sicherzugehen, dass die Testperson die Anweisung verstanden hat.)*  **1 – 2 – 2 – 1 – 2 – 1 – 1 – 2 – 2 – 1 – 2 – 1 – 1 – 2** | | | | | | | | | | | | | | | | | | | | | | | | | | | | | | | | | | |  |  |
| **Affekt** | | | | | | **Geben Sie 6 Punkte, wenn keine Auffälligkeiten vorliegen. Ziehen Sie 1 Punkt pro vorhandenes Item ab. „Nicht-Bestanden“ bei 4 Punkten oder weniger.**  *(Der Testleiter schätzt ein, ob die folgenden Auffälligkeiten vorliegen, Eindrücke von der Testperson oder deren engen Bezugspersonen werden miteinbezogen.)* | | | | | | | | | | | | | | | | | | | | | | | | | | | | | **/6** |  |
| [ ] Hat Schwierigkeiten die Aufmerksamkeit zu fokussieren oder bei der mentalen Flexibilität  [ ] Emotional labil, unpassende/inkongruente Emotionen, erscheint hoffnungslos oder depressiv  [ ] Zeigt schnell Zeichen einer Reizüberflutung oder ausweichendes Verhalten  [ ] Äußert unlogische Gedanken oder Paranoia  [ ] Zeigt fehlende Empathie, ist apathisch oder zeigt eine Affektverarmung  [ ] Ist zornig oder aggressiv, reizbar, konfrontativ, hat Schwierigkeiten mit sozialen Grenzen oder sozialen  Regeln/Normen | | | | | | | | | | | | | | | | | | | | | | | | | | | | | | | | | | |  |  |
| **GESAMTWERT** | | | | | | | | | | | | | | | | | | | | | | | | | | | | | | | | | | | **/120** | **/10** |
| **Berechnen Sie den Gesamtwert (1. Spalte) und den Gesamtwert der „Nicht-Bestandenen“ Aufgaben (2. Spalte).**  **1 „Nicht-Bestandene“ Aufgabe = mögliches CCAS; 2 „Nicht-Bestandene“ Aufgaben = wahrscheinliches CCAS; 3 oder mehr „Nicht-Bestandene“ Aufgaben = definitives CCAS.** | | | | | | | | | | | | | | | | | | | | | | | | | | | | | | | | | | | | |

| Hinweise und Mehrfachauswahl für die Aufgabe „Verbaler Abruf“ | | | | | |
| --- | --- | --- | --- | --- | --- |
| **Testwort** | **Dorf** | **Petra** | **Glück** | **antworten** | **viereckig** |
| **Hinweis** | Ort, an dem Personen leben | Frauenname | positive Emotion | kann man in Gesprächen tun | beschreibt die Form eines Objekts |
| **Mehrfach- auswahl** | Stadt | Sabine | Liebe | antworten | dreieckig |
|  | Ortschaft | Susanne | Glück | reagieren | rund |
|  | Dorf | Andrea | Genuss | erklären | oval |
|  | Vorort | Petra | Lachen | zuhören | viereckig |

| **Gemeinsamkeiten** | **Korrekte konzeptuelle Antwort (Beispiele)** | **Teilweise korrekte/konkrete Antwort (Beispiele)** |
| --- | --- | --- |
| Orange/Karotte | Lebensmittel | Saft machen, essen, Farbe |
| Schnecke/Krabbe | Tiere, Schale | klein, leben im Ozean |
| Schuhe/Gürtel | Kleidungszubehör | Leder, Farbe |
| Buch/Zeitung | Lesestoff, Information | Papier, Wörter |

**[Note: The German CCAS Scale has not been validated yet, and especially the cut-off values may change.]**

*Zeichnen Sie den Würfel hierhin ab.*

**Hoche, Guell, Vangel, Sherman, Schmahmann, Ataxia Center, Cognitive Behavioral Neurology Unit, Schmahmann Laboratory for Neuroanatomy and Cerebellar Neurobiology, Department of Neurology, Massachusetts General Hospital.**

**© 2016 The General Hospital Corporation. All Rights Reserved.**

**[Note: The German CCAS Scale has not been validated yet, and especially the cut-off values may change.]**

**CEREBELLAR COGNITIVE AFFECTIVE /
SCHMAHMANN SYNDROME Skala (CCAS-Skala) ID#: Bildungsjahre:
VERSION 1D. Datum:**

| **Semantische Wortflüssigkeit** | | | | | | | **Punkte = alle korrekten Wörter (maximal 26 Wörter). „Nicht-Bestanden“ bei 15 Punkten oder weniger.**  *(Nutzen Sie den Platz unten für Notizen.)* | | | | | | | | | | | | | | | | | | | | | | | | | | | | | **Punkte** | | **Bestanden= 0**  **Nicht-Bestanden= 1** | |
| --- | --- | --- | --- | --- | --- | --- | --- | --- | --- | --- | --- | --- | --- | --- | --- | --- | --- | --- | --- | --- | --- | --- | --- | --- | --- | --- | --- | --- | --- | --- | --- | --- | --- | --- | --- | --- | --- | --- | --- |
| Bitte nennen Sie so viele Möbelstücke oder Haushaltsgeräte wie Ihnen innerhalb einer Minute einfallen. | | | | | | | | | | | | | | | | | | | | | | | | | | | | | | | | | | | | **/26** | |  | |
| **Phonematische Wortflüssigkeit** | | | | | | | **Punkte = alle korrekten Wörter (maximal 19 Wörter). „Nicht-Bestanden“ bei 9 Punkten oder weniger.**  *(Nutzen Sie den Platz unten für Notizen.)* | | | | | | | | | | | | | | | | | | | | | | | | | | | | | **/19** | |  | |
| Bitte nennen Sie so viele Wörter wie möglich mit dem Anfangsbuchstaben *„M“* wie Ihnen innerhalb einer Minute einfallen. Benutzen Sie nicht Namen von Personen oder Orten und wiederholen Sie nicht dasselbe Wort in unterschiedlichen Formen. *(Unterschiedliche Formen desselben Wortes sind z.B. „der Fisch, des Fisches, die Fische“)* | | | | | | | | | | | | | | | | | | | | | | | | | | | | | | | | | | | |  |  |  |  |
| **Kategorie-Wechsel** | | | | | | | **Punkte = Anzahl der korrekten Wechsel zwischen den Wortkategorien (maximal 15 Wechsel). Wiederholungen und Regelbrüche werden nicht bewertet. „Nicht-Bestanden“ bei 9 Punkten oder weniger.** *(Nutzen Sie den Platz unten für Notizen.)* | | | | | | | | | | | | | | | | | | | | | | | | | | | | | **/15** | |  | |
| Bitte nennen Sie einen Männernamen und dann ein Tier, dann einen anderen Männernamen und dann wieder ein weiteres Tier usw. Wechseln Sie immer zwischen den beiden Kategorien ab. Nennen Sie so viele Wörter wie Ihnen innerhalb einer Minute einfallen. | | | | | | | | | | | | | | | | | | | | | | | | | | | | | | | | | | | |  |  |  |  |
| **Verbales Lernen** | | | | | | | **Diese Aufgabe wird nicht mit Punkten bewertet. (Wenn 4 Versuche benötigt werden, die 5 Wörter zu lernen, ist dies ein Hinweis für eine zerebrale Beteiligung.)** | | | | | | | | | | | | | | | | | | | | | | | | | | | | |  | |  | |
| Ich werde Ihnen nun eine Wortliste vorlesen, welche Sie bitte lernen sollen. Bitte wiederholen und merken Sie sich die Wörter. Ich werde Sie in einigen Minuten noch einmal bitten, diese Wörter wiederzugeben.  *(Lesen Sie die 5 Wörter in einer Geschwindigkeit von 1/Sekunde vor. Die Testperson wiederholt die Wörter einmal, dann wiederholt sie diese ein weiteres Mal. Wiederholen Sie die Wortliste bis die Testperson alle 5 Wörter erinnert. Brechen Sie nach 4 Versuchen ab.)* | | | | | | | | | | | | | | | | | | | | | | | | | | | | | | | | | | | |  |  |  |  |
|  | | [Berg] | | | | | | | | | [Paris] | | | | | | | | [Gewalt] | | | | | | [schlafen] | | | | | | | [laut] | | | |  |  |  |  |
| Versuch 1 | | [ ] | | | | | | | | | [ ] | | | | | | | | [ ] | | | | | | [ ] | | | | | | | [ ] | | | |  |  |  |  |
| Versuch 2 | | [ ] | | | | | | | | | [ ] | | | | | | | | [ ] | | | | | | [ ] | | | | | | | [ ] | | | |  |  |  |  |
| Versuch 3 | | [ ] | | | | | | | | | [ ] | | | | | | | | [ ] | | | | | | [ ] | | | | | | | [ ] | | | |  |  |  |  |
| Versuch 4 | | [ ] | | | | | | | | | [ ] | | | | | | | | [ ] | | | | | | [ ] | | | | | | | [ ] | | | |  |  |  |  |
| **Zahlenspanne Vorwärts** | | | | | | | **Punkte = längste korrekt wiederholte Zahlenspanne. „Nicht-Bestanden“ bei 5 Punkten oder weniger.** | | | | | | | | | | | | | | | | | | | | | | | | | | | | | **/8** | |  | |
| Ich werde Ihnen nun einige Zahlen jeweils einmal vorlesen. Bitte wiederholen Sie diese in der exakt gleichen Reihenfolge. *(Lesen Sie die Zahlen in einer Geschwindigkeit von 1/Sekunde laut vor. Beginnen Sie beim * und führen Sie die vorherigen Zahlenspannen nur durch, wenn * falsch wiedergegeben wurde.)* | | | | | | | | | | | | | | | | | | | | | | | | | | | | | | | | | | | |  |  |  |  |
| 9-2 | [ ] | | | 0-4-3-1* | | | | | | [ ] | | | | | 1-0-8-6-4-7 | | | | | | | [ ] | | | | | 3-5-2-1-7-9-8-4 | | | | | | | [ ] | |  |  |  |  |
| 7-8-5 | [ ] | | | 6-3-9-7-2 | | | | | | [ ] | | | | | 2-0-1-5-6-4-9 | | | | | | | [ ] | | | | |  | | | | | | |  | |  |  |  |  |
| **Zahlenspanne Rückwärts** | | | | | | | **Punkte = längste korrekt wiedergegebene Zahlenspanne. „Nicht-Bestanden“ bei 3 Punkten oder weniger. Können 2 Ziffern nicht korrekt rückwärts wiedergegeben werden, werden 0 Punkte vergeben.** | | | | | | | | | | | | | | | | | | | | | | | | | | | | | **/6** | |  | |
| Nun geben Sie die Zahlen bitte rückwärts wieder, also in umgekehrter Reihenfolge. *(Machen Sie das Beispiel vor, dann beginnen Sie beim *.)* | | | | | | | | | | | | | | | | | | | | | | | | | | | | | | | | | | | |  |  |  |  |
| Bsp.: 5-8 = 8-5 | | | *1-6 | | | | | [ ] | 0-8-3 | | | | [ ] | | | | 5-9-7-2 | | | | [ ] | | 8-3-1-6-4 | | | | | [ ] | 7-2-9-5-3-0 | | | | | | [ ] |  |  |  |  |
| **Würfel (Zeichnen)** | | | | | | | **Punkte = 15 Punkte, wenn 12 Linien gezeichnet wurden und die Zeichnung dreidimensional ist. Hat die Zeichnung nicht 12 Linien oder ist nicht dreidimensional, führen Sie die Aufgabe *„Würfel (Abzeichnen)“* durch.** | | | | | | | | | | | | | | | | | | | | | | | | | | | | | **/15** | |  | |
| Bitte zeichnen Sie einen sechsseitigen Würfel. Zeichnen Sie ihn transparent, also durchsichtig. *(Nutzen Sie den Platz auf Seite 2.)* | | | | | | | | | | | | | | | | | | | | | | | | | | | | | | | | | | | |  |  |  |  |
| **Würfel (Abzeichnen)** | | | | | | | **Punkte = 12 Punkte, 1 Punkt pro Linie. Ziehen Sie jeweils 1 Punkt ab, wenn die Zeichnung nicht dreidimensional ist, 1 Punkt für jede Linie, die nicht gezeichnet wurde und 1 Punkt für jede zusätzlich gezeichnete Linie > 12. „Nicht-Bestanden“ bei 11 Punkten oder weniger.** | | | | | | | | | | | | | | | | | | | | | | | | | | | | |  |  |  |  |
| Bitte zeichnen Sie den Würfel ab, der auf Seite 4 abgebildet ist. *(Sorgfalt/Genauigkeit wird nicht bewertet.)* | | | | | | | | | | | | | | | | | | | | | | | | | | | | | | | | | | | |  |  |  |  |
|  | | | | | | | | | | | | | | | | | | *Notizen:* | | | | | | | | | | | | | | | | | | | | | |
| Semantische WF | | | | | | | | | | | | | | Phonematische WF | | | | | | | | | | | | | | | | | Kategorie-Wechsel | | | | | | | | |
|  | | | | | |  | | | | | | | | | | | | | | | | | | | | | | | | | | | | | | |  | |  |
| \| **[Note: The German CCAS Scale has not been validated yet, and especially the cut-off values may change.]**  *Zeichnen Sie den Würfel hier.*  **[Note: The German CCAS Scale has not been validated yet, and especially the cut-off values may change.]** \|  \| \| --- \| --- \| | | | | | | | | | | | | | | | | | | | | | | | | | | | | | | | | | | | | | | | |
| **Verbaler Abruf** | | | | | | **Spontan = 3 Punkte pro Wort, Kategorie = 2 Punkte, Mehrfachauswahl = 1 Punkt. Punkte = Gesamtpunktzahl. „Nicht-Bestanden“ bei 10 Punkten oder weniger. Wird nicht mehr als ein Wort bei der Mehrfachauswahl erinnert, ist dies ein Hinweis für eine zerebrale Beteiligung.** | | | | | | | | | | | | | | | | | | | | | | | | | | | | | | | **Punkte** | | **Bestanden= 0**  **Nicht-Bestanden= 1** |
| Wie lauten die Wörter, die ich Sie vor einer Weile gebeten habe, zu lernen? *(Die Testperson gibt die Wörter, die sie sich vorher merken sollte, wieder. Benutzen Sie die Hinweise und die Mehrfachauswahl (unten), wenn notwendig.)* | | | | | | | | | | | | | | | | | | | | | | | | | | | | | | | | | | | | | **/15** | |  |
|  | | | | | [Berg] | | | | | | | [Paris] | | | | | | | | [Gewalt] | | | | | | [schlafen] | | | | | | | [laut] | | | |  |  |  |
| Spontane Wiedergabe | | | | | [ ] | | | | | | | [ ] | | | | | | | | [ ] | | | | | | [ ] | | | | | | | [ ] | | | |  |  |  |
| Mit Hinweis | | | | | [ ] | | | | | | | [ ] | | | | | | | | [ ] | | | | | | [ ] | | | | | | | [ ] | | | |  |  |  |
| Mit Mehrfachauswahl | | | | | [ ] | | | | | | | [ ] | | | | | | | | [ ] | | | | | | [ ] | | | | | | | [ ] | | | |  |  |  |
| **Gemeinsamkeiten** | | | | | | **Korrekte Antwort (konzeptuell) = 2 Punkte, teilweise korrekte Antwort (konkret) = 1 Punkt, inkorrekte/ keine Antwort = 0 Punkte. Punkte = Gesamtpunktzahl. „Nicht-Bestanden“ bei 6 Punkten oder weniger. Auswertungshilfe – unten und in der Durchführungsanleitung.** | | | | | | | | | | | | | | | | | | | | | | | | | | | | | | | **/8** | |  |
| Was haben die folgenden Wörter gemeinsam? Was ist ihre Gemeinsamkeit? *(Geben Sie ein Beispiel, testen Sie danach die Items.)* | | | | | | | | | | | | | | | | | | | | | | | | | | | | | | | | | | | | |  |  |  |
| Bsp.: Schaf/Elefant = Tiere | | | | | | | 1.Milch/Ei | | | | | | | | | 2.Hammer/  Schraubendreher | | | | | | | | 3.Seefahrer/  Pilot | | | | | | 4.Armband/Ohrring | | | | | | |  |  |  |
|  |  |  |  |  |  |  | [ /2] | | | | | | | | | [ /2] | | | | | | | | [ /2] | | | | | | [ /2] | | | | | | |  |  |  |
| **Go/No-Go** | | | | | | **2 Punkte für keinen Fehler, 1 Punkt für 1 Fehler, 0 Punkte für 2 oder mehr Fehler. Punkte = Gesamtpunktzahl. „Nicht-Bestanden“ bei 0 Punkten.** | | | | | | | | | | | | | | | | | | | | | | | | | | | | | | | **/2** | |  |
| Ich werde nun auf den Tisch klopfen. Wenn ich einmal klopfe, heben Sie bitte den Finger und senken Sie ihn wieder! Wenn ich zweimal klopfe, machen Sie bitte nichts! *(Geben Sie ein Beispiel für jede Bedingung um sicherzugehen, dass die Testperson die Anweisung verstanden hat.)*  **1 – 2 – 2 – 1 – 1 – 2 – 1 – 2 – 2 – 1 – 2 – 1 – 1 – 2** | | | | | | | | | | | | | | | | | | | | | | | | | | | | | | | | | | | | |  |  |  |
| **Affekt** | | | | | | **Geben Sie 6 Punkte, wenn keine Auffälligkeiten vorliegen. Ziehen Sie 1 Punkt pro vorhandenes Item ab. „Nicht-Bestanden“ bei 4 Punkten oder weniger.**  *(Der Testleiter schätzt ein, ob die folgenden Auffälligkeiten vorliegen, Eindrücke von der Testperson oder deren engen Bezugspersonen werden miteinbezogen.)* | | | | | | | | | | | | | | | | | | | | | | | | | | | | | | | **/6** | |  |
| [ ] Hat Schwierigkeiten die Aufmerksamkeit zu fokussieren oder bei der mentalen Flexibilität  [ ] Emotional labil, unpassende/inkongruente Emotionen, erscheint hoffnungslos oder depressiv  [ ] Zeigt schnell Zeichen einer Reizüberflutung oder ausweichendes Verhalten  [ ] Äußert unlogische Gedanken oder Paranoia  [ ] Zeigt fehlende Empathie, ist apathisch oder zeigt eine Affektverarmung  [ ] Ist zornig oder aggressiv, reizbar, konfrontativ, hat Schwierigkeiten mit sozialen Grenzen oder sozialen  Regeln/Normen | | | | | | | | | | | | | | | | | | | | | | | | | | | | | | | | | | | | |  |  |  |
| **GESAMTWERT** | | | | | | | | | | | | | | | | | | | | | | | | | | | | | | | | | | | | | **/120** | | **/10** |
| **Berechnen Sie den Gesamtwert (1. Spalte) und den Gesamtwert der „Nicht-Bestandenen“ Aufgaben (2. Spalte).**  **1 „Nicht-Bestandene“ Aufgabe = mögliches CCAS; 2 „Nicht-Bestandene“ Aufgaben = wahrscheinliches CCAS; 3 oder mehr „Nicht-Bestandene“ Aufgaben = definitives CCAS.** | | | | | | | | | | | | | | | | | | | | | | | | | | | | | | | | | | | | | | | |

| Hinweise und Mehrfachauswahl für die Aufgabe „Verbaler Abruf“ | | | | | |
| --- | --- | --- | --- | --- | --- |
| **Testwort** | **Berg** | **Paris** | **Gewalt** | **schlafen** | **laut** |
| **Hinweis** | etwas, das man besteigen/ beklettern kann | Städtename | ein negatives Verhalten | tut man, wenn man müde ist | beschreibt die Qualität eines Tons |
| **Mehrfach- auswahl** | Hügel | London | Grausamkeit | schlafen | weich |
|  | Leiter | Paris | Ärger | liegen | laut |
|  | Berg | Tokyo | Feindseligkeit | ausruhen | knallend |
|  | Baum | Amsterdam | Gewalt | Nickerchen machen | klopfend |

| **Gemeinsamkeiten** | **Korrekte konzeptuelle Antwort (Beispiele)** | **Teilweise korrekte/konkrete Antwort (Beispiele)** |
| --- | --- | --- |
| Milch/Ei | Lebensmittel, Rezeptzutaten | weiß, kühlen |
| Hammer/Schraubendreher | Werkzeuge | Metall, man hält diese, werden auf dem Bau benutzt |
| Seefahrer/Pilot | Berufe, navigieren Fahrzeuge | tragen eine Uniform |
| Armband/Ohrring | Schmuck, Accessoires | man trägt diese, glänzend, teuer |

**[Note: The German CCAS Scale has not been validated yet, and especially the cut-off values may change.]**

*Zeichnen Sie den Würfel hierhin ab.*

Hoche, Guell, Vangel, Sherman, Schmahmann, Ataxia Center, Cognitive Behavioral Neurology Unit, Schmahmann Laboratory for Neuroanatomy and Cerebellar Neurobiology, Department of Neurology, Massachusetts General Hospital.

© 2016 The General Hospital Corporation. All Rights Reserved.

**4. Instructions of the American English CCAS Scale translated to German**

**[Note: This paragraph is the direct translation of the American English version. The German CCAS Scale has not been validated yet, and the information given may change.]**

**Cerebellar Cognitive Affective / Schmahmann Syndrome (CCAS) Skala**

**Anleitung zur Durchführung und Bewertung**

Die „*Cerebellar Cognitive Affective/ Schmahmann Syndrome (CCAS)“ Skala* ist ein Screening-Instrument zur Erfassung des *zerebellären kognitiven affektiven Syndroms* bei Patienten mit zerebellären Läsionen. Es bewertet verschiedene kognitive Domänen: Aufmerksamkeit und Konzentration, exekutive Funktionen, Gedächtnis, Sprache, visuell-räumliche Fähigkeiten, abstraktes Denken, und neuropsychiatrische Aspekte. Die Durchführung der Skala dauert ca. 10 Minuten bei gesunden Testpersonen, und ca. 12 Minuten bei Patienten mit Defiziten. Der maximal erreichbare Punktwert beträgt 120 Punkte; maximal können 10 Aufgaben bestanden oder nicht bestanden werden. Ein Normalbefund ist definiert als das Bestehen aller 10 Aufgaben. Besteht ein Patient mit zerebellärer Erkrankung eine Aufgabe nicht, ist ein *CCAS möglich*. Werden 2 Aufgaben nicht bestanden ist ein *CCAS wahrscheinlich*. Werden 3 Aufgaben oder mehr nicht bestanden gilt ein *CCAS* als *gesichert*.

Dokumentieren Sie den Namen des Patienten, das Geburtsdatum und die Fallnummer. Vermerken Sie die Bildungsjahre des Patienten; 1 Jahr pro Schuljahr (Vollendung der 12. bzw. 13. Klasse = 12 bzw. 13 Schuljahre), und fügen Sie (falls zutreffend) weitere Bildungsjahre für Ausbildung oder Studium hinzu. Notieren Sie das Testdatum.

1. **Semantische (Kategoriale) Wortflüssigkeit**

Durchführung: Der Testleiter gibt der Testperson folgende Anweisung: „Bitte nennen Sie so viele Tiere oder Lebewesen wie Ihnen innerhalb einer Minute einfallen. Sind Sie bereit? Beginnen Sie jetzt!“

Bewertung: Vergeben Sie je richtiger Antwort innerhalb einer Minute einen Punkt.

Beispiel: Die Testperson antwortet: „Eule, Vogel, Fledermaus, Kuh, Gras, Käfer, Pferd, Hund“, ergibt 7 Punkte („*Gras“* ist ein Kategorienfehler).

Folgende zählen als richtige Antwort:

- Verschiedene Bezeichnungen/ Geschlechter für ähnliche Tiere, z.B. Stute, Hengst, Henne, Hahn
- Kategorien und Beispiele aus den Kategorien, z.B. Hund, Pudel, Cavallier; Vogel, Adler, Kardinal; Fisch, Lachs, Forelle
- Aufzählung ausgestorbener Tierarten, z.B. Dinosaurier, Pterodactylus (Flugsaurier)

Folgende sind falsche Antworten und zählen nicht:

- Fehler wie *„Blume“* anstelle von Tieren oder Lebewesen
- Wiederholungen des gleichen Wortes
- Grammatikalische Beugungen des gleichen Wortes – Elefant, Herde von Elefanten; Hund, Hunde; roter Vogel, blauer Vogel, gelber Vogel

1. **Phonematische (Lexikalische) Wortflüssigkeit**

Durchführung: Der Testleiter gibt der Testperson folgende Anweisung: „Bitte nennen Sie so viele Wörter wie möglich mit dem Anfangsbuchstaben *„F“* wie Ihnen innerhalb einer Minute einfallen. Benutzen Sie nicht Namen von Personen oder Orten und wiederholen Sie nicht dasselbe Wort in unterschiedlichen Formen. Sind Sie bereit? Beginnen Sie jetzt!“

Bewertung: Vergeben Sie je richtig genanntem Wort innerhalb einer Minute einen Punkt. Fehler werden nicht gezählt (z.B., wenn der Proband *„Vogel“* nennt, anstelle eines Wortes mit dem Anfangsbuchstaben *„F“*). Dasselbe gilt für Namen von Personen oder Orten und für verschiedene Formen desselben Wortes (z.B. *„der Fisch, des Fisches“*, hingegen sind z.B*. „der Fisch, das Fischen“* korrekte Worte, da sie verschiedene Bedeutungen haben). Wiederholungen desselben Wortes werden nicht gezählt.

Beispiel: Die Testperson antwortet: „Feder, Feuer, Freund, Franz, Freiheit, Feder, Fernseher, Fernglas, Freunde.“, ergibt 6 Punkte. *(„Franz“* ist ein Regelverstoß, *„Feder“* eine Wiederholung, *„Freunde“* ist eine andere Form eines bereits genannten Wortes).

1. **Kategorie-Wechsel (Gemüse-Beruf)**

Durchführung: Der Testleiter gibt der Testperson folgende Anweisung: „Bitte nennen Sie eine Gemüsesorte und dann einen Beruf oder einen Job, dann eine andere Gemüsesorte und dann wieder einen weiteren Beruf usw. Wechseln Sie immer zwischen den beiden Kategorien ab. Nennen Sie so viele Wörter wie Ihnen innerhalb einer Minute einfallen. Sind Sie bereit? Beginnen Sie jetzt!“

Bewertung: Vergeben Sie je korrektem ***Wechsel zwischen den Kategorien*** innerhalb einer Minute einen Punkt. Die Wechsel zwischen den Kategorien werden gezählt und die Wörter der Kategorien müssen stimmen (und dürfen keine Wiederholungen sein). Fehler, die nicht sofort von der Testperson selbst korrigiert werden, werden nicht gezählt. Zum Beispiel sind Kategorienfehler wie *„Apfel“* anstelle einer Gemüsesorte falsch und werden nicht gezählt. Wenn das erste Wort in den Kategorien falsch ist (z.B. keine Gemüsesorte, kein Beruf), dann unterbrechen Sie und vergewissern Sie sich, dass die Testperson die Anweisung versteht. Danach beginnen Sie von vorn. Wenn die Testperson nach den ersten Worten drei Fehler hintereinander gemacht hat, können Sie diese erinnern, was die Kategorien waren, aber beginnen Sie die Aufgabe nicht von vorn. Es werden nur die Wechsel zwischen Wörtern aus den richtigen Kategorien mit Punkten bewertet.

Beispiele:

| Gurke |  | 1 |
| --- | --- | --- |
| Arzt |  | 2 |
| Sellerie |  | 3 |
| Postbote |  | 4 |
| Lauch |  | 5 |
| Lehrer |  |  |
| *Apfel* |  |  |
| Bibliothekar |  | 6 |
| Spinat |  |  |
| *Postbote* |  |  |
| Brokkoli |  | 7 |
| Sekretärin |  |  |

Apfel wird nicht gezählt (Kategorienfehler). Postbote wurde zweimal wiederholt (Wiederholung). Dadurch ist der Gesamtpunktwert 7.

1. **Verbales Lernen**

Durchführung: Der Testleiter gibt der Testperson folgende Anweisung: „Ich werde Ihnen nun eine Wortliste vorlesen, welche Sie bitte lernen sollen. Bitte wiederholen und merken Sie sich die Wörter. Ich werde Sie in einigen Minuten noch einmal bitten, diese Wörter wiederzugeben. Sind Sie bereit? Das sind die Wörter.“

(Lesen Sie die 5 Wörter in einer Geschwindigkeit von 1/Sekunde vor. Lassen Sie die Testperson die Wörter wiedergeben, sobald Sie die Wörter vorgelesen haben. Lassen Sie die Testperson nach Aufforderung noch einmal die Wörter wiederholen. Wiederholen Sie dieses Vorgehen bis die Testperson alle 5 Wörter wiedergeben kann, aber stoppen Sie nach 4 Versuchen. Die Testperson kann die fünf Wörter in jeder Reihenfolge wiedergeben. Dokumentieren Sie, wie viele Wörter wiederholt werden konnten.)

Bewertung: Dieser Teil der CCAS Skala wird nicht mit Punkten bewertet. Wenn 4 Versuche benötigt werden, die 5 Wörter zu lernen, ist dies ein Hinweis für eine zerebrale Beteiligung.

1. **Zahlenspanne Vorwärts**

Durchführung: Der Testleiter gibt der Testperson folgende Anweisung: „Ich werde Ihnen nun einige Zahlen jeweils einmal vorlesen. Bitte wiederholen Sie diese in der exakt gleichen Reihenfolge. Sind Sie bereit? Das sind die Zahlen.“

(Lesen Sie laut mit einer Geschwindigkeit einer Ziffer/Sekunde vor. Beginnen Sie mit * (4 Ziffern). Wenn die Testperson 4 Ziffern nicht in der korrekten Reihenfolge wiedergeben kann, versuchen Sie 3, und dann 2. Wenn die Testperson 4 Ziffern richtig wiederholt, dann lesen Sie die 5-Ziffernfolge, dann die 6-Ziffernfolge usw. Stoppen Sie, wenn Sie die 8-Ziffernfolge erreicht haben, oder wenn die Testperson die Wiederholung nicht schafft. Erlauben Sie einen Versuch pro Ziffernfolge.)

Bewertung: Vergeben Sie einen Punkt pro Ziffer für die längste Ziffernfolge, die korrekt wiedergegeben wurde. Jeder Fehler in einer Ziffernfolge (z.B. Testperson sagt *„1-6-9-****4****-5“* anstelle von *„1-6-9-****2****-5“)*, der nicht sofort von der Testperson selbst korrigiert wird, zählt als Fehler, und die längste zuvor korrekt wiedergegebene Ziffernfolge wird zur Bewertung herangezogen (hier: 4 Punkte, nicht 5).

1. **Zahlenspanne Rückwärts**

Durchführung: Der Testleiter gibt der Testperson folgende Anweisung: „Nun geben Sie die Zahlen bitte rückwärts wieder, also in umgekehrter Reihenfolge. Wenn ich „5-8“ sage, sollen Sie „8-5“ sagen. Haben Sie die Aufgabe verstanden? Ich werde jede Zahlenfolge nur einmal vorlesen. Sind Sie bereit? Das sind die Zahlen.“

(Starten Sie mit *(2 Ziffern). Stoppen Sie, wenn Sie die 6-Ziffernfolge erreicht haben, oder wenn die Testperson die Ziffernfolge nicht korrekt rückwärts wiedergeben kann. Erlauben Sie einen Versuch pro Ziffernfolge.)

Bewertung: Vergeben Sie einen Punkt pro Ziffer für die längste Ziffernfolge, die korrekt rückwärts wiedergegeben wurde. Jeder Fehler in einer Ziffernfolge (z.B. *„****8-2****-3“* anstelle von *„****2-8****-3“)*, der nicht sofort von der Testperson selbst korrigiert wird, zählt als Fehler, und die längste zuvor korrekt rückwärts wiedergegebene Ziffernfolge wird zur Bewertung herangezogen.

1. **Würfel (Zeichnen)**

Durchführung: Der Testleiter gibt der Testperson folgende Anweisung: „Bitte zeichnen Sie einen sechsseitigen Würfel. Zeichnen Sie ihn transparent, also durchsichtig.“

(Keine Zeitvorgabe. Sorgfalt/Genauigkeit wird nicht bewertet.)

Bewertung: Vergeben Sie maximal 15 Punkte, wenn alle 12 Linien vorhanden sind und die Zeichnung dreidimensional ist. Wenn < 12 Linien oder > 12 Linien vorhanden sind und der Würfel nicht dreidimensional ist, führen Sie die Aufgabe *„Würfel (Abzeichnen)“* durch. Dann werden keine Punkte für die Aufgabe *„Würfel (Zeichnen)“* vergeben.

Beispiele:

| 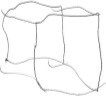 | Korrekte Zeichnung (hat 12 Linien und ist dreidimensional). Die Linien müssen nicht perfekt gezeichnet sein. |
| --- | --- |
| 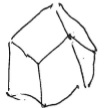 | Fehlerhafte Zeichnung (hat 13 Linien und ist nicht dreidimensional).  Führen Sie die Aufgabe „*Würfel (Abzeichnen)*“ durch.  (Wenn dieser Würfel bei der Aufgabe *„Würfel (Abzeichnen)“* gezeichnet worden wäre, hätte er mit 10 Punkten bewertet werden müssen. 12-1 für die zusätzliche Linie; -1 dafür, dass die Zeichnung nicht dreidimensional ist.) |
| 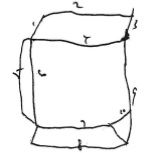 | Fehlerhafte Zeichnung (hat 14 Linien; 2 Linien mehr als erlaubt und ist nicht dreidimensional).  Führen Sie die Aufgabe *„Würfel (Abzeichnen)“* durch.  (Wenn dieser Würfel bei der Aufgabe *„Würfel (Abzeichnen)“* gezeichnet worden wäre, hätte er mit 9 Punkten bewertet werden müssen. 12-2 für die zusätzlichen Linien; -1 dafür, dass die Zeichnung nicht dreidimensional ist.) |

Der Patient sollte den Würfel so gut zeichnen, wie es ihm möglich ist. Beobachten Sie, ob der Patient dieses versucht. Ungenauigkeiten aufgrund von Schwierigkeiten in der Kontrolle des Stifts zählen nicht als Fehler. Der Test misst die visuell-räumliche Konzeptplanung und -ausführung durch Bewertung der Fähigkeit des Patienten 12 Linien zu zeichnen und die Zeichnung dreidimensional erscheinen zu lassen. Der Test ist nicht konzipiert, um die motorische Kontrolle zu messen. Wenn der Patient den Würfel nicht korrekt zeichnen kann, dann lassen Sie ihn den Würfel kopieren. Wenn der Patient aufgrund einer Dysmetrie der oberen Extremität zu beeinträchtigt ist, um den Stift zu halten, oder ihn stabil genug zu halten, um den Würfel zu zeichnen oder abzuzeichnen, dann schließen Sie diese Aufgabe nicht in den Gesamtpunktwert ein. In diesem Fall läge der Gesamtpunktwert bei 105, nicht bei 120. Ebenso, wird in diesem Fall, der Test nicht als „nicht-bestanden“ (in der „Bestanden“/“Nicht-Bestanden“-Spalte) gezählt. Notieren Sie, dass die Dysmetrie zu stark war, um diesen Test durchzuführen.

1. **Würfel (Abzeichnen)**

Durchführung: Der Testleiter gibt der Testperson folgende Anweisung: „Bitte zeichnen Sie den Würfel ab, der auf Seite 4 abgebildet ist.“ (Keine Zeitvorgabe. Sorgfalt/Genauigkeit wird nicht bewertet.)

Bewertung: Vergeben Sie maximal 12 Punkte – 1 Punkt für jede gezeichnete Linie. Ziehen Sie jeweils einen Punkt ab, wenn der Würfel nicht dreidimensional ist, und für jede fehlende Linie (< 12 Linien) oder zusätzliche Linie (> 12 Linien).

Beispiele:

| 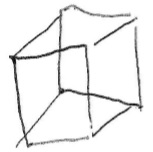 | Korrektes Abzeichnen. Vergeben Sie 12 Punkte. |
| --- | --- |
| 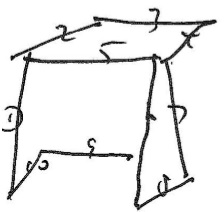 | Fehlerhaftes Abzeichnen. Vergeben Sie 10 Punkte (10 Punkte für jede gezeichnete Linie, dreidimensionale Zeichnung wurde versucht, kein weiterer Punktabzug). |
| 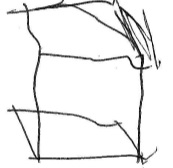 | Fehlerhaftes Abzeichnen. Vergeben Sie 10 Punkte (10 Punkte für jede gezeichnete Linie, dreidimensionale Zeichnung wurde versucht, kein weiterer Punktabzug; kein Punktabzug für die motorischen Schwierigkeiten). |

1. **Verbaler Abruf**

Durchführung: Der Testleiter gibt der Testperson folgende Anweisung: „Wie lauten die Wörter, die ich Sie vor einer Weile gebeten habe, zu lernen?“

(Die Testperson gibt die Wörter, die sie sich vorher merken sollte, wieder. Benutzen Sie die Hinweise und die Mehrfachauswahl (unten), wenn notwendig.)

Bewertung: Spontanes Wiedergeben wird mit 3 Punkten pro Wort bewertet. Wiedergeben nach Nennen eines Hinweises wird mit 2 Punkten pro Wort bewertet. Wiedergeben nach Nennen der Mehrfachauswahl wird mit 1 Punkt pro Wort bewertet.

Beispiel: Eine Testperson gibt die folgenden Wörter mit den dargestellten Hilfen wieder und erhält eine Gesamt-Punktzahl von 6+2+1 = 9 Punkten.

| **Spontane Wiedergabe** | **Blume**  [X] | **Frank**  [ ] | **Mut**  [ ] | **sprechen**  [ ] | **gelb**  [X] | **Gesamt**  [6] |
| --- | --- | --- | --- | --- | --- | --- |
| **Mit Hinweis** | [ ] | [X] | [ ] | [ ] | [ ] | [2] |
| **Mit Mehrfachauswahl** | [ ] | [ ] | [X] | [Nicht wiedergegeben nach Mehrfachauswahl] | [ ] | [1] |

1. **Gemeinsamkeiten**

Durchführung: Der Testleiter gibt der Testperson folgende Anweisung: „Was haben die folgenden Wörter gemeinsam? Was ist ihre Gemeinsamkeit?“

(Geben Sie ein Beispiel, testen Sie danach die Items.)

Beachten Sie: Wenn die Testperson eine Antwort gibt, die teilweise richtig ist (mit **Q** markiert), dann fragen Sie: „Können Sie eine genauere (konzeptuelle) Antwort geben, was die beiden Begriffe gemeinsam haben?“ – Wenn die Testperson jetzt eine 2-Punkte-Antwort gibt, vergeben Sie 2 Punkte.

Bewertung: Richtige (bestmögliche) = konzeptuelle Antwort (2 Punkte), teilweise richtige = konkrete Antwort, aber nicht bestmögliche Antwort = 1 Punkt, falsche Antwort oder keine Antwort = 0 Punkte.

Bewertungsschlüssel und Beispielantworten:

***Dieser Bogen sollte dem Testleiter während der Testung vorliegen.***

Nase-Ohr

| 2 Punkte | Sinne (werden hierfür benutzt, sind Teil der) Sinne  Sinnes (-organe; -teile; -punkte)  Organe, die genutzt werden, um Dinge wahrzunehmen  Zwei der fünf Sinne |
| --- | --- |
| 1 Punkt | Gesichts (-teile, -merkmale); (Teile von, in, an) Deinem Gesicht **(Q)**  Merkmale von Säugetieren  Körperteile, Teile des Körpers **(Q)**  (Teile von, an) Deinem Kopf |
| 0 Punkte | Liefern dem Körper Geruch und Gehör **(Q)**  Man kann durch Nase und Mund atmen  Gesicht; Kopf (**Q)**  helfen Dir beim Atmen und Hören (oder ein anderer Unterschied) |

Schaf-Elefant

| 2 Punkte | Tiere; Säugetiere; Pflanzenfresser  Mitglieder (des Tierreiches; der Gattung der Tiere)  Vierbeiner |
| --- | --- |
| 1 Punkt | Beide haben (vier Beine; einen Schwanz), haben vier Beine und einen Schwanz (Namen und physische Merkmale, die beide gemeinsam haben) **(Q)**  Können gezähmt werden  Beide sind (stark, kräftig, muskulös, schnell) |
| 0 Punkte | Man sieht sie im Zoo (Zirkus, woanders)  Sie gehören zu derselben Spezies  Sind wild; leben in der Wildnis  Werden in der Natur gefunden  Einer hat Wolle, der andere hat eine dicke Haut  Einer ist groß, der andere ist klein  Oder andere Unterschiede |

See-Fluss

| 2 Punkte | Gewässer, Wasser |
| --- | --- |
| 1 Punkt | (Beide sind) kalt, nass **(Q)**  Du kannst in ihnen schwimmen (spielen, trainieren) **(Q)**  Du kannst sie trinken **(Q)** |
| 0 Punkte | Beide sind blau  Einer ist groß, der andere klein  Einer ist lang, der andere rund  Einer steht still, der andere fließt  Oder andere Unterschiede |

Flugzeug-Motorrad

| 2 Punkte | (Mittel, Formen, Arten) der/ des Transport(s); beide transportieren Menschen  (Mittel, Formen, Arten) der Reise/des Reisens; zum Reisen, zur Reise  Fahrzeuge, Beförderungsmittel  Eine Art von einem zum anderen Ort zu gelangen  Sie bringen Dich (Orte, irgendwohin); sie befördern Dich an ein Ziel |
| --- | --- |
| 1 Punkt | Man fährt beide **(Q)**  Beide bewegen sich (fort) **(Q)**  Beide werden zum Vergnügen und zur Erholung genutzt  Beide überbrücken Distanzen  Sie müssen (gesteuert, gefahren, bedient werden) **(Q)**  Man fährt, steuert, bedient sie **(Q)**  Sie befördern (Leute, Dinge) **(Q)** |
| 0 Punkte | Beide haben (Motoren, Triebwerke, Sitze, Lenkräder, oder andere gemeinsame Details) **(Q)**  (fahren mit, benötigen) (Treibstoff, Benzin) **(Q)**  Sie sind teuer  Beide sind mechanisch **(Q)**  Ein Flugzeug ist für die Luft, ein Motorrad ist für die Straße  Das Flugzeug hat Flügel/fliegt, ein Motorrad hat Räder/fährt  (Oder andere Unterschiede) |

1. **Go/No-go**

Durchführung: Der Testleiter gibt der Testperson folgende Anweisung: „Ich werde nun auf den Tisch klopfen. Wenn ich einmal klopfe, heben Sie bitte Ihren Finger und senken Sie ihn wieder! Wenn ich zweimal klopfe, machen Sie bitte nichts! Hier sind zwei Beispiele, um sicherzugehen, dass Sie verstehen, was ich meine. (Zur Demonstration einmal bzw. zweimal auf den Tisch klopfen.) Sind Sie bereit? Los geht‘s!“

(Die Intervalle zwischen den Klopf-Bedingungen sollen 1 Sekunde betragen. Wird zweimal auf den Tisch geklopft, so sollen die beiden Tischberührungen sehr schnell hinter einander erfolgen (innerhalb von Millisekunden), damit die Testperson sicher zwischen den beiden Klopf-Bedingungen unterscheiden kann.)

Bewertung: Notieren Sie sich Fehler und Auslassungen. 0 Fehler = Vergeben Sie die maximalen 2 Punkte. 1 Fehler oder Auslassen = vergeben Sie 1 Punkt. 2 oder mehr Fehler = vergeben Sie 0 Punkte.

1. **Affekt**

Durchführung: Der Testleiter beobachtet das Verhalten und die Interaktion der Testperson während des Tests. Der Testleiter beurteilt, ob die Verhaltensweisen, die auf dem Bewertungsbogen aufgeführt sind, vorhanden sind. Zur Bewertung können Ergänzungen durch den Patienten oder nahe Bezugspersonen herangezogen werden, die der Testleiter auf das Vorliegen dieser Symptome befragt.

Bewertung: Vergeben Sie 6 Punkte, wenn keine der aufgelisteten Verhaltensweisen vorhanden ist. Ziehen Sie für jede Verhaltensweise, die vorhanden ist, einen Punkt ab.

Beispiel: Wenn die Aussage *„Hat Schwierigkeiten die Aufmerksamkeit zu fokussieren oder bei der mentalen Flexibilität“* auf die Testperson zutrifft, aber keine der anderen Verhaltensweisen vorhanden ist, werden 6-1 = 5 Punkte vergeben.

Hoche, Guell, Vangel, Sherman, Schmahmann, Ataxia Center, Cognitive Behavioral Neurology Unit, Schmahmann Laboratory for Neuroanatomy and Cerebellar Neurobiology, Department of Neurology, Massachusetts General Hospital. © 2016 The General Hospital Corporation. All Rights Reserved.

**5. References (Supplements)**

1. Hoche, F., Guell, X., Vangel, M.G., Sherman, J.C., & Schmahmann, J.D. (2018). The cerebellar cognitive affective/Schmahmann syndrome scale. *Brain,*  *141(1)*, 248-270. doi: 10.1093/brain/awx317.

2. Kessler, J., Bley, M., Mielke, R., & Kalbe, E. (1997). Strategies and structures in verbal fluency tasks in patients with Alzheimer's disease. *Behav Neurol,*  *10(4)*, 133-135. doi: 10.3233/BEN-1997-10406.

3. Kalbe, E., Kessler, J., Calabrese, P., Smith, R., Passmore, A.P., Brand, M., & Bullock, R. (2004). DemTect: a new, sensitive cognitive screening test to support the diagnosis of mild cognitive impairment and early dementia. *Int J Geriatr Psychiatry,*  *19(2)*, 136-43. doi: 10.1002/gps.1042.

4. Delis, D.C., Kaplan, E., & Kramer, J.H. (2001). *Delis-Kaplan Executive Function System®(D-KEFS®): Examiner's Manual: Flexibility of Thinking, Concept Formation, Problem Solving, Planning, Creativity, Impluse Control, Inhibition.* San Antonio, TX: The Psychological Cooperation.

5. Wechsler, D. (2008). *Wechsler adult intelligence scale–Fourth Edition (WAIS–IV).* San Antonio, TX: NCS Pearson.

6. Kokmen, E., Naessens, J.M., & Offord, K.P. (1987). A short test of mental status: description and preliminary results. *Mayo Clin Proc,*  *62(4)*, 281-288. doi: 10.1016/s0025-6196(12)61905-3.

7. Guell, X., Hoche, F., & Schmahmann, J.D. (2015). Metalinguistic deficits in patients with cerebellar dysfunction: empirical support for the dysmetria of thought theory. *Cerebellum,*  *14(1)*, 50-58. doi: 10.1007/s12311-014-0630-z.

8. Nasreddine, Z.S., Phillips, N.A., Bedirian, V., Charbonneau, S., Whitehead, V., Collin, I., Cummings, J.L., & Chertkow, H. (2005). The Montreal Cognitive Assessment, MoCA: a brief screening tool for mild cognitive impairment. *J Am Geriatr Soc,*  *53(4)*, 695-699. doi: 10.1111/j.1532-5415.2005.53221.x.

9. Daly, M., Sherman, J.C., & Schmahmann, J.D. (2016). The cerebellar neuropsychiatric rating scale (CNRS): development of a new assessment tool for the affective component of the CCAS (abstract). *J Neuropsychiatry Clin Neurosci,*  *28(3)*, 41-42. doi: 10.1176/appi.neuropsych.15080210.

10. Costa, A.S., Fimm, B., Friesen, P., Soundjock, H., Rottschy, C., Gross, T., Eitner, F., Reich, A., Schulz, J.B., Nasreddine, Z.S., & Reetz, K. (2012). Alternate-form reliability of the Montreal cognitive assessment screening test in a clinical setting. *Dement Geriatr Cogn Disord,*  *33(6)*, 379-384. doi: 10.1159/000340006.
